# The Impact of Limited English Proficiency on Healthcare Access and Outcomes in the U.S.: A Scoping Review

**DOI:** 10.3390/healthcare12030364

**Published:** 2024-01-31

**Authors:** Sylvia E. Twersky, Rebeca Jefferson, Lisbet Garcia-Ortiz, Erin Williams, Carol Pina

**Affiliations:** 1 Department of Public Health, The College of New Jersey, Ewing Township, NJ 08618, USA; ortizl3@tcnj.edu (L.G.-O.);; 2 R. Barbara Gitenstein Library, The College of New Jersey, Ewing Township, NJ 08618, USA; jeffersr@tcnj.edu

**Keywords:** limited English proficiency, immigrants, hospitalizations, ambulatory care, screening, healthcare outcomes, healthcare costs, mental health, health disparities

## Abstract

A majority of individuals with limited English proficiency (LEP) in the U.S. are foreign-born, creating a complex intersection of language, socio-economic, and policy barriers to healthcare access and achieving good outcomes. Mapping the research literature is key to addressing how LEP intersects with healthcare. This scoping review followed PRISMA-ScR guidelines and included PubMed/MEDLINE, CINAHL, Sociological Abstracts, EconLit, and Academic Search Premier. Study selection included quantitative studies since 2000 with outcomes specified for adults with LEP residing in the U.S. related to healthcare service access or defined health outcomes, including healthcare costs. A total of 137 articles met the inclusion criteria. Major outcomes included ambulatory care, hospitalization, screening, specific conditions, and general health. Overall, the literature identified differential access to and utilization of healthcare across multiple modalities with poorer outcomes among LEP populations compared with English-proficient populations. Current research includes inconsistent definitions for LEP populations, primarily cross-sectional studies, small sample sizes, and homogeneous language and regional samples. Current regulations and practices are insufficient to address the barriers that LEP individuals face to healthcare access and outcomes. Changes to EMRs and other data collection to consistently include LEP status and more methodologically rigorous studies are needed to address healthcare disparities for LEP individuals.

## 1. Introduction

### 1.1. Background 

Language differences are a critical barrier to healthcare access as well as healthcare quality and effective outcomes. The U.S. Department of Justice defines limited English proficiency (LEP) as individuals who do not speak English as their primary language and who have a limited ability to read, speak, write, or understand English [1]. However, the medical literature often frames this differently since if electronic medical records capture LEP at all, it is often only in the form of the primary language spoken. Because of this, research studies looking at LEP use a range of definitions for determining LEP populations. 

The primary language spoken may also be used as a proxy indicator for immigrant status, which is not often captured in medical data, despite immigrants being a vulnerable population for poor health outcomes due to reduced access to primary care [2,3,4]. Immigration patterns shape the LEP population in the U.S., as the majority of LEP individuals are foreign-born [5]. In the United States, 8.4% of households spoke English less than very well in 2022. The proportion of the population with LEP in the U.S. varies by language group, with the largest LEP population among Spanish speakers. According to the 2022 American Community Survey, 5.3% of Spanish speakers have LEP. The language groups with the second highest percentage are Chinese and Indo-European language speakers (0.6%), then other Asian and Pacific language speakers (0.4%), followed by Vietnamese and Russian, Polish, or other Slavic speakers (0.3%), and French, Haitian, or Creole, Korean, Tagalog, and Arabic speakers (0.2%), and lastly German or other West Germanic language speakers (0.1%) [6]. Linguistic diversity within the U.S. population, as well as the complex intersection of language with healthcare access, patient-provider communication, and socio-economic barriers, necessitate an in-depth examination of how LEP influences healthcare outcomes. 

LEP individuals may encounter challenges accessing high-quality healthcare services, potentially leading to delays in care, medical errors, difficulty understanding and following provider directions, and other stumbling blocks to good health outcomes. Even the magnitude of the effect of adverse events can be greater for LEP populations, as evidenced by the fact that medical errors experienced by LEP individuals were more likely to cause physical harm compared to those experienced by patients who spoke English [7]. Recognizing this disparity, Title VI of the Civil Rights Act of 1964 was determined by the Supreme Court to cover LEP individuals and ensure that they are not discriminated against, and the Office for Civil Rights has the authority to investigate complaints and even withhold federal funds related to linguistic access [8]. In 2000, renewed attention was given to Title VI with Executive Order 13166, Improving Access to Services for Persons with Limited English Proficiency. This federal policy required all healthcare providers that received federal funds, including Medicare and Medicaid, to examine their policies and assess them for meaningful access to services by LEP individuals [9]. While this and other federal laws explicitly require access to language services such as interpretation and translation of documents in healthcare, a lack of knowledge and enforcement leave many LEP individuals without access to these key services. There are a few states that have large LEP populations, including California, Texas, Florida, and New York [10]. Because of this impact, these and other states have enacted their own supplemental statutes and regulations that try to help clarify and broaden access to quality healthcare for LEP individuals. As of 2019, every state in the U.S. has at least three provisions regarding language access in healthcare, although the specifics vary from comprehensive laws to (more commonly) laws that address only specific populations, providers, or healthcare modalities. California has the most comprehensive legislation, with 257 provisions in effect [11]. LEP populations can face significant disparities in health outcomes due to linguistic barriers and their intersection with other barriers, such as lack of health insurance and discrimination. Many LEP populations are also people of color, with LEP further exacerbating barriers many racial/ethnic groups already experience in the United States. For example, the LEP population in the U.S. is three times as likely as the English-proficient population to be uninsured [10].

### 1.2. Rationale for the Current Study

Some studies have shown that LEP is associated with poor asthma outcomes and higher healthcare resource utilization [12,13,14]. Multiple studies show that LEP is independently associated with lower use of preventive healthcare, poorer health behaviors linked to chronic disease, and a longer length of hospital stay for diabetes diagnoses [15,16,17]. However, the literature on hospital utilization is mixed, showing that the LEP population has an overall lower number of Emergency Department visits due to preventable causes such as asthma and diabetes than their English-proficient counterparts [18], is less likely to be admitted to the hospital for ambulatory-sensitive conditions from the ED [19], and has lower rates of diagnosed chronic disease [15]. The disparities among outcomes may occur because studies often focus on one specific geographic region within the U.S. and/or specific language groups and their experiences with the healthcare system. It then becomes even more important to understand and map the research literature in terms of LEP definitions, healthcare settings, geographic regions studied, linguistic groups that are included/excluded, and outcomes that are assessed in order to make sense of differences in the literature and potential gaps for LEP experiences and outcomes within the multimodal healthcare system. 

### 1.3. Objectives

This review aims to provide a critical overview of the role that limited English proficiency (LEP) can play in determining healthcare outcomes across multiple domains in the U.S. healthcare system, including disease screening, ambulatory care access and outcomes, hospital utilization and outcomes, general health measures, and outcomes for specific conditions. Our review will focus on adults in a U.S. context and on spoken language. While definitions of LEP and healthcare literacy certainly can and do include written language, practically speaking, U.S. electronic medical records typically contain only information regarding the primary language spoken; therefore, it makes sense to keep this focus in our chosen context. Because we aim to map the literature on this topic and identify gaps, the scoping review methodology is the best fit for our objectives. By synthesizing insights from previous research, we can identify gaps in current knowledge and contribute to the research-based policy and practice decisions made to address healthcare disparities related to linguistic barriers.

## 2. Methods

### 2.1. Literature Sources and Search Strategy

An overall procedure was developed by the PI and study team in cooperation with the librarian author, following PRISMA-ScR guidelines. Please see supplementary materials for the PRISMA-ScR checklist. Our search strategy was designed to cover a broad range of healthcare modalities, looking specifically at health outcomes, and as such, included the clinical research literature. Clinically focused databases included were PubMed/MEDLINE and CINAHL. PubMed/MEDLINE was chosen over other medical databases because of its update frequency and inclusion of early online articles, as well as its focus on medicine [20], while CINAHL adds to the search an emphasis on the perspectives of nursing and allied health disciplines. In addition, searches were conducted in ProQuest’s Sociological Abstracts and EBSCO’s EconLit and Academic Search Premier. Database selection was designed to provide as broad a range of coverage as possible, including the medical, economic, and social science literature. Test searches were conducted and were used to develop lists of search terms tailored to each database. These search term lists included MeSH terms, other database-specific subject terms, and other term combinations agreed upon by the study team based on the selection criteria. Every effort was made to maintain consistency in the search terms; however, some variations proved necessary in order to properly adapt our objectives to the differing controlled vocabularies used across these heterogeneous subject databases in order to obtain specific and relevant search results. Final searches were conducted throughout June 2023. For details of search strings adapted to individual databases and associated results, see Table 1.

### 2.2. Study Selection

As described above, we systematically searched the following electronic databases: PubMed/MEDLINE, CINAHL, Sociological Abstracts, EconLit, and Academic Search Premier. Searches took place in June 2023. In addition to database searches, the team performed hand searches of select literature reviews as well as related article searches using relevant database tools. Study selection was performed following PRISMA-ScR guidelines.

We included primary studies that met the following criteria:

1. Included adults with limited English proficiency as a clearly defined subset of the study population. We defined adults as subjects over the age of 18.
2. Included LEP subjects residing in the United States. We excluded studies not conducted in the United States, as policies and laws that govern language services as well as access to care may be different across English-speaking countries.
3. Study outcomes were quantitative and related to healthcare service access (e.g., health screenings, ambulatory care, hospital care, or mental health) or to clearly defined health outcomes, including outcomes related to healthcare costs.
4. Quantitative health care outcomes were specified for LEP populations based on spoken language. As noted in our objectives, electronic medical records typically contain only information regarding the primary language spoken. In order to isolate the effects of spoken language from those of literacy, we focused on studies that had outcomes based on spoken language only or clearly defined a subset of results based on spoken English ability.

We excluded studies for the following reasons:

1. Because our objective was to focus on spoken language proficiency among immigrant populations that have a primary language other than English, we excluded any studies primarily focused on a deaf or hard-of-hearing population. Although this is an important topic, the research team felt that it would require a separate and focused review.
2. We excluded studies with outcomes focused on access to particular drugs or types of drugs (such as opiate pain medications). Studies that assessed specific interventions were also excluded. Studies with only qualitative outcomes were excluded from the analysis.
3. Studies published prior to 2000 were excluded by database filters due to changes in policy around medical records data collection and language accessibility. Our searches included dissertations and theses. However, we elected to exclude conference presentations, posters, and preprints. We excluded studies written in languages other than English and studies not primarily focused on adults, defined as subjects ages 18 and older.
4. Studies were excluded if healthcare outcomes were not specified for the LEP population, if the LEP definition was not clearly based only on spoken language, or if outcomes were not available for spoken language only.

**Figure 1:**
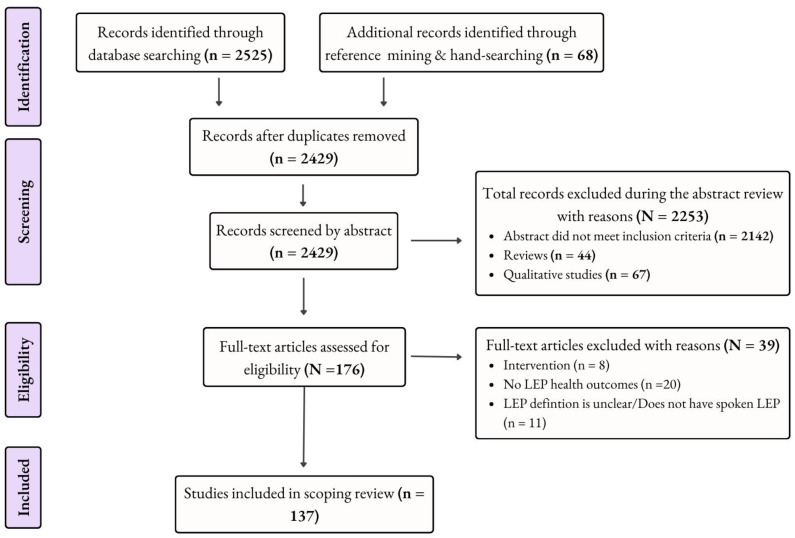
PRISMA flow diagram describing the scoping review search for studies examining the impact of limited English proficiency (LEP) on adult healthcare services utilization and outcomes in the U.S.

Following database searches, all identified citations were collated and uploaded into Zotero, and duplicates were removed. Our review team then conducted pilot testing with 20% of the abstracts to assess inter-reviewer agreement on inclusion/exclusion criteria. We assessed inter-reviewer agreement in the pilot screening based on percent agreement in order to ensure that the inclusion and exclusion criteria were clear and applied consistently. Based on the high percent agreement (94.5%) in the pilot screening, all abstracts were screened using the inclusion and exclusion criteria provided above by members of our review team, and a process of consensus was used for any exclusions. Any abstract exclusions were agreed upon by at least two team members. The remaining potentially relevant sources were retrieved in full-text form and assessed in detail against the inclusion criteria by members of the study team. Reasons for the exclusion of full-text studies that did not meet the inclusion criteria were recorded. All exclusions at the full-text stage were discussed and agreed upon by at least three members of the review team, including the PI. Any disagreements that arose at each stage of the selection process were resolved through full team discussion. For further details of the search flow, please see Figure 1 below.

### 2.3. Data Extraction

For each study, we extracted the following data points: Primary health-related outcome; study design; study period (years); study setting or context; how the LEP population was defined for the study; both the total sample size and the LEP sample size; languages spoken by participants (if recorded); results related to the LEP sample studied and associated effect sizes; bibliographic information. Because secondary data that identifies immigrant populations is difficult to find, the team additionally identified any secondary datasets that were specified as part of the research. While generally scoping reviews do not include a critical appraisal of the sources of evidence, we made broad determinations of study quality based on objective study characteristics, including sample size, research methodology, and generalizability of the study, in order to add context to the study outcomes. During data extraction, the studies were categorized by major outcomes, which included ambulatory care outcomes, hospitalization outcomes, screening outcomes (that is, studies looking at screening initiation and access to screening tests), specific condition outcomes, and general physical and mental health outcomes. If a research study had outcomes that fit into more than one category, the study and specific outcomes that fit the outcome category were included in each applicable category, so that studies that addressed more than one outcome category could be included multiple times in the results section.

## 3. Results

After review, 137 studies met the inclusion criteria. Table 2 provides an overview of publicly available secondary datasets that were used for various studies, listing outcome categories, in order to allow researchers interested in LEP and healthcare to more easily identify data that can be used to understand this at-risk population. Major themes of the results by outcome category are summarized below, and Table 3, Table 4, Table 5, Table 6 and Table 7 provide specific data on study methodology, sample size, linguistic groups, and study outcomes.

### 3.1. Ambulatory Care Studies

There were 29 studies looking at the influence of LEP on ambulatory care outcomes that met our inclusion criteria after review [2,21,22,23,24,25,26,27,28,29,30,31,32,33,34,35,36,37,38,39,40,41,42,43,44,45,46,47,48]. A majority of studies had a cross-sectional design, and many used large national datasets to conduct analysis, such as the National Health Interview Survey and the Medical Expenditure Panel Survey. A regional survey that was frequently utilized was the California Health Interview Survey. The two main ethnicities researched were Asian and Hispanic populations, and this is likely the reason why those studies that identified languages spoken by LEP participants included mostly Spanish and Asian languages. However, many studies did not specify languages spoken, only participant ethnicities. All of the studies in this group were low to moderate in terms of quality due to small sample sizes that may not be representative of the population, unclear or potentially inaccurate definitions of LEP, and the general lack of data on LEP populations due to a greater focus on acculturation, ethnicities, and/or literacy. This resulted in a scarcity of LEP-specific ambulatory outcome data in many of the included studies. 

The majority of the ambulatory care studies looked at healthcare utilization and access. These studies found that LEP patients were significantly less likely than their English-proficient (EP) counterparts to have a regular source of care and that EP individuals had a greater number of physician visits compared to LEP individuals [2,21,23,26,27,28,33,36,38,39,41,42,43,44,47]. Most studies found that LEP individuals were more likely to forgo necessary medical care, less likely to receive preventive care, less likely to have a usual source of care, and even, in one case, showed an increase in missed preventive care visits [36] compared to EP populations. Two studies, Njeru et al. and Pylypchuk et al., found mixed results on access to care. Njeru et al. looked at adherence to telephone line triage recommendations for healthcare and found that LEP patients were less likely to follow through with recommendations to call an ambulance, visit the emergency room, and recommend home care, but more likely to follow through with a routine visit within a week compared to the EP population [49]. Pylypchuk et al. found a mix of outcomes looking at preventive care for LEP immigrants compared to their native counterparts depending on insurance type. The analysis showed that LEP immigrants with private insurance were less likely to have their flu shot, have their cholesterol checked, go to the dentist, and have a breast exam in the past year, but there was no difference in medical visits. However, for individuals who had public insurance or were uninsured, there were no significant differences in preventive care between LEP immigrant and native populations [30]. There were two studies in this category that either did not find a significant difference between LEP and EP populations in their ability to access ambulatory care [31] or that LEP populations had higher rates of seeing a primary care physician and/or specialty care physician compared to EP populations [37]. This study also found that LEP populations had lower rates of emergency room visits. Because of the differences in ambulatory care utilization found by the majority of studies, Himmelstein et al. found that Hispanic LEP individuals spend USD 1463 less overall on medical care compared to their Hispanic EP counterparts and USD 2802 less than non-Hispanic EP individuals [39]. Jacobs et al. found that the use of interpreter services increased the number of clinical (ex: office and urgent care visits, phone calls, and prescriptions) and preventive services (ex: mammograms, fecal occult blood testing, rectal exams, and flu vaccinations) received among LEP patients [40]. This indicates that changes to service provision could ameliorate differences in access and outcomes for LEP patients. 

There were three ambulatory care studies that specifically looked at mental health services [22,25,33]. For all three studies, having LEP significantly reduced the likelihood of utilizing mental health services for some or all of the populations studied. The studies focused on Hispanic only [33] or Hispanic and Asian [22,25] populations. Bauer et al. found that both Hispanic and Asian LEP populations were less likely to access mental health services over their lifetimes and spent more time living with untreated mental illness compared to the EP population. Meanwhile, Kim et al. looked specifically at immigrant populations with psychiatric disorders and found that LEP significantly affected mental health service use for the Hispanic population but had no statistically significant effect on the Asian population with psychiatric disorders. Two of the three mental health studies had small sample sizes, and all three studies were cross-sectional.

The rest of the included studies investigated different specific aspects of ambulatory care. One study looked at the likelihood of LEP individuals completing an advanced directive and found that the probability of advanced directive completion among Hispanic LEP individuals was much lower than their EP counterparts. It was also found that living in a Spanish-speaking community was a negative predictor of advanced directive completion [34]. Another study looked at cardiovascular risk reduction outcomes in a pharmacist-managed clinic and found no significant difference between LEP and EP groups but had a very small sample size for LEP (n = 9) [35]. Studying a refugee population, Geltman et al. found significantly lower rates of both overall dental care and specifically preventive visits for LEP refugees compared to their EP counterparts [48]. Another study looked at medication management in home healthcare and found that LEP patients showed less improvement in both oral and injectable medication management with home healthcare [45]. Even when looking at specific ambulatory care services rather than general access and outcomes, the majority of studies found that service use, process of care, and outcomes showed significant disparities between LEP and EP populations. Please see a list of studies included in this category with key summary information in Table 3 below.

**Table 3:** Ambulatory Care Study Details.

Ambulatory Care Citation	Health Outcome	Study Design	Study Period	Sample Size (N=)	LEP Sample Size (n=)	Setting	LEP Definition	Languages	Study Outcomes (LEP Related)
[25]	mental health service use	cross-sectional	2002–2003	1147	465	United States	poor/fair English speaking ability	Spanish, Mandarin, Cantonese, Vietnamese, and Tagalong	Significantly fewer LEP individuals for both Latino and Asian populations (compared to EP individuals) accessed lifetime mental health services (42.8% vs. 54.2%, *p* = 0.01, 32.9% vs. 53.9%, *p* = 0.01). LEP individuals for both Latino (14.6 vs. 9.4 years, *p* = 0.01) and Asian populations (16.3 vs. 9.0 years, *p* = 0.001) live longer with their disorder untreated. The EP population had a significantly higher odds of lifetime treatment for their mental health, with EP Latinos and Asians (OR 1.7; OR 2.3) significantly more likely to receive treatment compared to LEP individuals.
[26]	usual source of care	cross-sectional	2005	2740	NA	California	reported speaking English less than ‘‘well.’’	NA	44.7% of LEP participants had a usual source of care other than the ER, significantly less than their EP counterparts (*p* < 0.01).
[31]	healthcare access	cross-sectional	2011–2019	5032	NA	Greater Los Angeles area	English speaking ability was described as not well or not at all	NA	No significant interaction between English proficiency and regular doctor access.
[33]	health care utilization (mental and physical health)	cross-sectional	1996–1997	31,003	1652	United States	language of survey	Spanish	For LEP Hispanic participants, 61% had a physician visit in the past year and 4.0% had a mental health visit in the past year. LEP Hispanic patients were significantly less likely than non-Hispanic White patients to have had a physician visit (RR, 0.77; CI, 0.72–0.83) and a mental health visit (RR, 0.50; CI, 0.32–0.76).
[48]	dental care	cross-sectional	2009	439	247	Massachusetts	Those who score no/low on the BEST Plus test	NA	Dental visit rates in the last year for LEP refugees were 48.6%, significantly lower than EP (*p* = 0.04). In the last year, 27.4% of LEP refugees had a preventative dental visit, significantly lower than EP (*p* < 0.01).
[34]	advanced care planning	cross-sectional	2013–2017	620,948	15,656	Northern California integrated health system	Needing an interpreter	Spanish	Advanced directive (AD) completion probability was much lower among Hispanic Spanish speakers compared to their English-speaking and White counterparts. Negative predictors of AD completion included living in a primarily Spanish- speaking community (living in a census tract where >35% of residents were Spanish speakers, OR = 0.9; 95% CI= 0.8–0.9).
[35]	cardiovascular risk outcomes	retrospective cohort study	2010–2012	71	9	Wishard Health Services & Eskenazi Health Indianapolis, IN	only speak Spanish	Spanish	There was no significant difference found in outcomes between the English speaking and Spanish speaking groups.
[37]	health service utilization	cross-sectional	2000	1703	565	Washington	Used interpreter service at least once	Spanish and other	A higher proportion of LEP patients visited primary care (95% versus 82%) and specialty care (60% versus 50%), but a lower proportion visited the emergency room (31% versus 47%). Annualized numbers of visits to primary care sites were 6.2 per year for LEP subjects compared to 3.8 for English speakers. Specialty visits were 2.9 per year for LEP subjects compared to 2.2 for English speakers.
[38]	usual source of care and healthcare utilization	cross-sectional	2018	21,177	1730	California	Participants who reported speaking English not well or not at all	Spanish, Cantonese, Mandarin, Korean, Tagalog, and Vietnamese	LEP individuals were significantly less likely than their EP counterparts to have a usual source of care other than the ER, have a usual place to go when sick or needing medical advice, have preventative care in the last year, delay not getting medical care in the past 12 months, and forgo necessary care (*p* < 0.01).
[39]	health care spending and utilization	cross-sectional	1998–2018	120,546	17,776	United States	if their interview was conducted in Spanish	Spanish	LEP Hispanics spent $1463 less on medical expenses on average compared to their EP Hispanic counterparts (*p* < 0.001). LEP Hispanic individuals spent $2802 less on medical expenses on average compared to EP non-Hispanic individuals (*p* < 0.001). LEP Hispanics spent $456 less on outpatient care on average compared to their EP Hispanic counterparts (*p* < 0.001). LEP Hispanic individuals spent $708 less on outpatient care on average compared to EP non-Hispanic individuals (*p* < 0.001). LEP individuals were significantly less likely to utilize outpatient visits compared to their non-Hispanic and Hispanic EP counterparts (*p* < 0.001).
[41]	healthcare utilization	cross-sectional	2006–2007	2884	NA	United States	English proficiency below moderate (read at least a little or somewhat)	NA	37.25% of those who were classified as LEP had used healthcare in the last 2 years compared to their proficient (81.20%) and moderately proficient (64.53%) counterparts (*p* < 0.001).
[42]	usual source of care	cross-sectional	2014	342	286	California	Participants who reported speaking English less than ‘very well’	Korean and other	Participants with LEP were 8.13 times more likely to not have no usual source of care (CI 2.40–27.56, *p* < 0.01).
[40]	delivery of healthcare & receipt of clinical and preventative health services	retrospective cohort study	1995–1997	4380	327	four HMOs in New England	Use of interpreter services	Spanish & Portuguese	There was a significant increase in nearly all clinical service usage (office visits, phone calls, urgent care visits, prescriptions written, and prescriptions filled) in the interpreter services group after the updated interpreter services were implemented. For example, there was a greater increase in the number of prescriptions filled by those in the interpreter services group (2.33 prescriptions per person) compared to those in the comparison group (0.86). For preventative services receipt (mammograms, breast exams, pap smears, fecal occult blood (FOB) testing, rectal exams, and flu vaccinations), the increase in receipt of these services in the control group ranged from 0.01–0.10. In contrast, the increase for the interpreter services group ranged from 0.01–0.26. There were significant increases in the number of rectal exams for men over 40 years old. However, this difference was not significant after adjusting for demographic differences between the groups.
[43]	usual source of care	cross-sectional	2015	2594	1618	Austin, Texas	Reported that they spoke English less than very well	NA	After controlling for covariates, the risk of having no usual place for care was 2.09 (42.2% vs. 31.4%) times higher among the LEP population (*p*< 0.001). The risk of having no regular check-up was 1.69 (35.6% vs. 27%) times as great (p < 0.001). Perceived unmet needs for medical care were 1.89 (14.6% vs. 6.4%) times as great (*p* < 0.001). Reported communication problems in healthcare settings were 4.95 (42.1% vs. 6.9%) times as great ( *p* < 0.001).
[36]	missed primary care appointments	cross-sectional	2015–2018	159,054	42,030	Hospitals in Boston	Preferred language of care was other than English	Spanish, Portuguese, and Haitian Creole	At baseline, the proportion of missed appointments was 19.4% among Spanish, Portuguese, and Haitian Creole speakers compared to 20.4% of English speakers. The prevalence of missed appointments increased by 0.74 percentage points (CI: 0.34, 1.15) among Spanish, Portuguese, and Haitian Creole speakers compared to English speakers over the same time period. This amounted to 799 additional missed appointments in the post-period than expected.
[44]	health care utilization	cross-sectional	2005–2007	217	NA	Arizona	low English speaking, writing, and reading skills	NA	Increased EP scores revealed a 6% increase in physician visits (*p* < 0.05).
[22]	mental health service use	cross-sectional	2002–2003	372	234	United States	Fair/poor English speaking ability	Spanish, Vietnamese, Mandarin, Cantonese, and	For adult Latino immigrants with psychiatric disorders, having LEP significantly decreased the odds of using mental health services (OR = 0.30; CI = 0.14, 0.64) compared to all immigrants with psychiatric disorders. In the Asian immigrant population with psychiatric disorders, LEP did not significantly affect mental health service use.
[21]	healthcare utilization	cross-sectional	2007	1745	988	California	English speaking ability was not well or not at all	Spanish, Korean, Mandarin, Vietnamese, and Cantonese	Among Asians, LEP individuals were less likely (*p* < 0.001) than EP and English only individuals to see a medical doctor in the past 12 months. Among Asian LEP population who had seen a doctor, the total number of doctor visits was significantly higher (5.73) than for EP (3.92) and English only (2.85). Group differences were not significant in the Latino population.
[23]	healthcare access	cohort cross- sectional	2006–2016	190,698	16,484	United States	(a) reported that a language other than English was spoken in their home or (b) reported that they did not speak English well or that they were not comfortable speaking English.	NA	The proportion of individuals with LEP who had a usual source of care before the ACA was 45.3% and after the ACA was 53.1% which was a significant improvement (*p* < 0.001). Compared to their LEP counterparts, EP individuals had a 4.9% higher chance of having a usual source of care (*p* < 0.001). The proportion of individuals with LEP who had to forgo any necessary care was reduced from 10.3% before the ACA to 7.1% after the ACA, a −3.5% difference. Compared to their LEP counterparts, EP individuals were 3.2% less likely to forgo any necessary care (*p* < 0.001). The proportion of individuals with LEP who had to forgo any necessary medical care was reduced from 4.8% to 2.8%, a −2.2% difference (*p* < 0.001). Compared to their LEP counterparts, EP individuals were 1.4% less likely to forgo necessary medical care. The proportion of individuals with LEP who had to forgo any necessary dental care was 7.5% before the ACA to 5.2% after the ACA, a −2.4% difference (*p* < 0.001).
[24]	dental care	cross-sectional	2013–2014		2114	United States	self-reported as “limited”	NA	When accounting for acculturation factors, LEP was a significant factor for having a dental visit in the previous 12 months (*p* < 0.05). However, after considering dental insurance and income, the LEP variable became insignificant (*p*= 0.18).
[45]	medication management with home healthcare	retrospective matched case- control	2010–2014	73,815 for oral; 7807 for injectable	17,662 fororal; 2248 for injectable	nonprofit home health agency	NA	NA	LEP was associated with less improvement in oral MM (.049, CI [.032-.065]) and injectable medications (.078, CI [.023-.133]) when compared to English-speaking patients.
[46]	eye care	cross-sectional	2000–2003	5455	2775	La Puenta, California	Preferring Spanish/Speaking only Spanish at home	Spanish	For participants who only spoke Spanish at home, the odds ratio for one or more eye care visits in the last 12 months was 0.79 (*p* < 0.05) compared to those who spoke English or both languages at home. There was no significant difference for having a dilated eye exam and having one or more dilated eye exams in the past 12 months.
[47]	usual source of care	cross-sectional	2003 and 2005	3011	1207	California	English speaking described as not well or not at all	N/A	10% of those with LEP had no usual source of care (*p* < 0.001, OR = 2.3).
[32]	following recommendations for healthcare visit	retrospective cohort study (chart review)	2012–2013	1174	587	Minnesota primary care practice	using an interpreter for the phone line	Somali, Asian languages -including Vietnamese, Cambodian and	LEP callers were less likely to follow the nurse’s recommendation than non-LEP callers (AOR, 0.65; *p* < 0.001). Stratified by recommended action LEP patients were less likely to follow through with recommendations to call an ambulance or visit the ED (AOR, 0.28; CI, 0.13, 0.60) and recommended home care (AOR, 0.34; CI, 0.22, 0.55), but more likely for follow through with a routine visit within a week (AOR, 2.45; 95% CI, 1.24, 4.82).
[2]	usual source of care, delays in getting care	cross-sectional	2001	18,000	1242	California	Individual reported speaking English not well or not at all	Spanish, Cantonese, Mandarin, Korean, Vietnamese, and Khmer,	In bivariate analysis LEP older adults had significantly higher proportions that lacked a usual source of care than older adults who speak English only. In multivariate analysis, LEP older adults had increased risk of not having a usual source of care (RR = 1.86, *p* = *0*.033) compared with English only speakers, but no significant differences in delays in care.
[30]	preventative care utilization	cross-sectional	2000–2004	NA	NA	United States	If respondent answered survey in another language besides English	NA	LEP immigrant population with private insurance was significantly less likely to get their flu shot, have their cholesterol checked, go to the dentist, and get a breast exam in the past year compared to the native population. There was no significant difference found for primary care visits, mammograms, or prostate exams between LEP immigrant population and native population with private insurance. There were no significant differences found in preventive care between immigrants with LEP and native populations with pubic insurance or who were uninsured.
[29]	primary care utilization	cross-sectional	NA	275	102	Tennessee	Speaks English a little or not at all	NA	There was no significant difference in LEP and EP individuals in visiting their primary care provider regularly (*p* =0.057).
[27]	delayed medical care, forgone needed care, and visits to healthcare professional	cross-sectional	2006	29,868	2606	United States	Speaks English less than very well	Spanish and other languages	Compared to English-proficient individuals, more individuals with LEP had forgone care (*p* < 0.05) and fewer reported healthcare visits (*p* < 0.001). Through unadjusted analyses, the study found that LEP individuals had 18% higher odds of forgoing medical care and 58% lower odds of having a healthcare visit compared to English-proficient individuals. In adjusted analyses, LEP individuals had 34% lower odds of having a healthcare visit.
[28]	healthcare utilization	cross-sectional	2000	49,327	NA	California, Colorado, Hawaii, Kansas, Michigan, New York, Ohio,	Survey language and language spoken at home were Spanish	Spanish and other	For participants who were Hispanic-Spanish and Asian-Other, there were significantly lower reports of timeliness of care, provider communication, and staff helpfulness (HS: −11.470, −3.575, −5.502, AO: −12.649, −7.158, −10.270; *p* < 0.001). There was also a significant difference in getting the care needed among Asian-Other participants(−8.459; *p* < 0.001).

NA means not available in the published manuscript. Abbreviations used in the table include the following: LEP, limited English proficiency; EP, English proficient.

### 3.2. Hospital Care Studies

There were 31 studies investigating limited English proficiency within hospital care settings that met our inclusion criteria [7,49,50,51,52,53,54,55,56,57,58,59,60,61,62,63,64,65,66,67,68,69,70,71,72,73,74,75,76,77,78]. More than half (18 in total) were retrospective cohort studies, the next most common being cross-sectional studies (seven in total). These hospital care studies addressed a variety of elements related to hospital care, such as end-of-life and palliative care, healthcare service delivery and patient satisfaction, interpreter impact, potentially preventable conditions, discharge instructions, length of stay, and hospital stay cost. Eight studies were conducted using data from California public hospitals or healthcare systems [50,51,52,53,54,55,56,57]. State and national datasets used in hospital care studies included the National Trauma Registry of the American College of Surgeons (NTRACS), the Agency for Healthcare Research and Quality’s (AHRQ) Healthcare Cost and Utilization Project (HCUP), California State Inpatient Databases, the American Hospital Association’s (AHA) Annual Survey, Hawaii’s Health Information Corporation’s (HHIC) inpatient database, and the Asian American Elders in New York City Study (AAENYC). The majority of the hospital care studies were considered moderate in terms of quality, primarily due to small LEP sample sizes, deficient LEP definitions, a lack of diversity in languages studied, and limited study locations. The most commonly studied language populations included Spanish, Cantonese, Mandarin, Portuguese, Arabic, and Russian, though this varied depending on location or dataset usage. A study by Sentell, Chang, Ahn, and Miyamura using the HHIC examined birth outcomes among a diverse range of Asian and Pacific Islander languages, such as Micronesian, Tagalong, Ilocan, Visayan, Chuukese, Marshallese, Tongan, and Samoan [58]. One study by Hines et al. using HCUP investigated inpatient mortality rates and obstetric trauma among 20+ languages [40]. There were five hospital care studies that did not specify languages and used a broad LEP categorization. These five studies examined emergency department outcomes and readmissions [59,60], unintentional adverse events [7], opioid discharge pain management in trauma patients [57], and emergency medical services scene and transport times [61].

A key area of interest among the hospital care studies was length of stay (LOS) and readmissions. In some studies, LOS was significantly greater for LEP populations, such as palliative care patients [62] and those who underwent a total joint arthroplasty [63]. In one study, LEP status was significantly associated with an overall increased hospital LOS for traumatic injuries when compared to EP status, though intensive care unit (ICU) LOS was shorter among LEP patients [50]. Increased LOS patterns for LEP patients were not found in studies looking at non-trauma-related hospital stays [53,64]. The impact of interpreter usage on LOS was inconsistent across hospital departments. While two studies found that use of interpreter services decreased in-hospital LOS [65,66], Wallbrecht et al. found that LOS from time of arrival to discharge in an emergency department (ED) increased when interpreters were used [67]. Studies exploring hospital admission and readmission had more consistent findings. Six studies found that LEP patients were more likely to be admitted upon visiting the emergency department or to be readmitted within 30 days [49,56,59,60,68,69,70], and another study determined that interpreter usage minimized the likelihood of 30-day readmission [65]. 

Another area of interest in hospital care studies was post-discharge understanding of care. Studies showed that EP patients tended to be prescribed more medications at discharge [57,71], specifically more opioids, and the level of post-discharge understanding of care was consistently lower among patients with LEP compared to EP. LEP patients were often unaware of their prescribed medications’ purpose and required more assistance with filling their prescriptions [71,72], though another study highlighted that understanding increased with interpreter assistance [73]. Despite having an initial lower level of post-discharge understanding, one study found that LEP patients asked more questions regarding their treatment, even without an interpreter, when compared to EP patients. This was particularly true for Spanish-speaking patients, who asked more clinical questions and reported issues with their discharge instructions more frequently in comparison to other LEP patients [72]. 

There were four hospital care studies that investigated the impact of LEP status on gynecological and obstetrics service delivery and outcomes. While one study found that LEP status was associated with an increased risk of having a primary cesarean delivery, particularly among those with diabetes [58], Hessol et al. found that LEP was significantly associated with lower rates of cesarean delivery compared to the EP population in one of the hospitals investigated but found no significant difference in outcomes at the other hospital in the study [51]. Hines et al. found that Spanish speakers and those who spoke Asian-Pacific Islander languages showed higher obstetric trauma rates when compared to EP patients [52]. LEP status could be a barrier for gynecological and obstetric Spanish-speaking patients who require pain medication if interpreter services are not always available. LEP patients who rarely received interpreter services during their hospitalization reported that healthcare professionals did not provide sufficient pain control, did not respond to patient needs in a timely manner, and were overall unhelpful when compared to LEP patients who always had consistent access to interpreter services during their stay [74]. This speaks to the contextual nature of LEP barriers to obstetric care, with adequate interpreter services potentially providing an ameliorating effect on outcome disparities.

The remaining hospital care studies primarily focused on healthcare service delivery and outcomes, as well as patient satisfaction. Several of these studies continued to examine the impact of interpreter services. In one study, English speakers tended to be more satisfied with their triage experiences, while Spanish speakers felt as if nurses did not understand their medical complaints [75]. In a different study, English-speaking patients received more tests and medical procedures than their LEP counterparts [76], though another study found that LEP patients were more likely to have electrocardiograms (ECG) performed by emergency medical services and within emergency departments [61]. Divi et al. conducted a study within six Joint-Commission-accredited U.S. hospitals showing that LEP patients experienced more unintentional adverse health events that resulted in physical harm not attributed to their initial condition or diagnosis compared to EP patients [79]. Finally, though LEP status was not associated with differences in advanced care planning discussions in hospitals [54], it was shown to impact informed consent. EP patients were more aware of informed consent, and interpreter services enhanced informed consent knowledge among LEP patients [55,77]. Differences in outcomes by hospital department, specifically differences across inpatient and ED settings, were shown consistently across multiple hospital-focused studies, indicating that LEP may impact care differently depending on the context. Importantly, significant disparities driven by LEP were identified in terms of processes such as informed consent and outcomes such as adverse health events. Please see a list of studies included in this category with key summary information in Table 4 below.

**Table 4:** Hospital Care Study Details.

Hospital Care Citations	Health Outcome	Study Design	Study Period	Total Sample Size (N=)	LEP Sample Size (n=)	Setting	LEP Definition	Languages	Study Outcomes (LEP Related)
[68]	end-of-life and palliative care	retrospective cohort	2010–2018	18,490	1363	Washington	“In what language do you want to talk to your healthcare team about your care?”	Mandarin Cantonese Vietnamese Russian Spanish	In adjusted analyses, LEP patients had higher odds of ED visits in the last 30 days (OR 1.47; CI 1.26, 1.72) & 180 days of life (OR 1.36; CI 1.17, 1.57). LEP patients had higher odds of 30-day readmission within the last 90-days (12% vs. 7.6%; OR 1.64, CI 1.30, 2.07) & 180-days of life (14.1% vs. 9.6%; OR 1.44; CI 1.16, 1.71) & higher odds of having an in-hospital death (OR 1.24; CI 1.07, 1.44). LEP patients had lower odds of advance care planning documents prior to death (OR 0.68; CI 0.59–0.80) when compared to EP patients.
[75]	door-to-room time and patient satisfaction	prospective cohort	2011–2013	163	55	Level 1 trauma center with an ED	Patients rated their language skills	Spanish	The median door-to-room and likelihood of admission was not significantly different between English-speakers and Spanish-speakers. English-speakers generally felt that the nurses completely understood their medical complaints, scoring a median of 5 on a 5 point Likert scale. Spanish speakers felt nurses mostly understood their medical complaint, scoring a median of 4 on a 5 points, and this comparison was statistically different between the groups. Spanish- speaking patients were significantly less satisfied with their triage experience than English-speaking patients. Of patients who described themselves English speakers, nurses misclassified one patient as having LEP. Of the patients who described themselves as Spanish speakers, nurses misclassified 15 as English speakers.
[78]	Potentially preventable intubations	retrospective cohort	1994–2003	274	21	Level 1 trauma center in eastern North Carolina	Patient’s primary language	Spanish	21 Spanish-speaking patients were intubated for less than 48 hours, compared to the 38% English-speaking patients. Spanish-speaking patients had less serious injuries as per the Injury Severity Score (ISS) compared to the English speaking group (10.5 vs. 13.0). The Spanish speaking group had greater Glasgow Coma Score (GCS) than English- speaking patients.
[62]	code status, advance directives, limiting life support decisions	retrospective cohort	2011–2014	27,523	779	Seven ICUs of varying specialties in a single center	Primary language or interpreter use as noted on medical chart	Arabic, Spanish, Somali, Cambodian, Vietnamese, Lao, Hmong, Russian,	After adjusting for illness severity, sex, education, & insurance status, patients with LEP were less likely to change their code status from full code to do not resuscitate (DNR) during ICU admission (OR, 0.62; *p* < 0.001) People with LEP who died in the ICU were less likely to receive a comfort measures order set (OR, 0.38; *p* = 0.03).
[76]	healthcare service delivery for initial ED visit and following 90 days	retrospective cohort	1999	500	437	urban academic teaching hospital	Self-reported primary language and if the patient is comfortable communicating in English	Spanish, Haitian Creole, and Portuguese Creole	English-speaking patients spent more hours (mean = 11.83 h, 95% CI 9.59–14.08) than LEP patients who did not receive interpreter services (8.62 hours, 95% CI 7.68–9.61) and LEP patients who did receive interpreter services (9.51 hours, 95% CI 7.10–11.92). English-speaking patients also had the highest post index visit ED cost (USD 988) when compared to the those who received interpreting services (USD 878) and those who did not (USD 710). English-speaking patients had more test and procedures done (mean = 13.40) than those who received interpreting services (12.69) and LEP patients who did not receive interpreting services (10.58). More English-speaking patients returned to the ED within 30 days of discharge (mean = 8724) than LEP patients who received interpretation services (7584) and those who did not receive interpreter services (5305).
[50]	morbidity and mortality after traumatic injury	retrospective cohort study	2012–2018	13,104	2144	Zuckerberg San Francisco General Hospital (ZSFG), an	English was not among patient self-reported languages spoken	Chinese, Spanish, and Other	LEP patients had an increased rate of TBI when compared to EP patients (41% versus 38%). In multivariate analyses, LEP patients were significantly associated with increased hospital LOS, decreased ICU LOS, decreased transfer to acute care hospital, and increased discharge home with home health services or skilled nursing facility (SNF)/rehabilitation.
[69]	healthcare utilization, end-of-life and palliative care for COVID-19 patients	retrospective cohort	2020	337	89	Two academic & four community hospitals in Boston	Self-reported primary language other than English listed in the EHR	Creole, Russian, Portuguese, Italian, Cantonese, Vietnamese, Portuguese Creole, Khmer, French,	More LEP patients died in the ICU than EP patients (61.8% vs. 35.1%). More LEP patients received CPR when compared to EP patients (10.1% vs. 3.6%). Patients with LEP were admitted or transferred to the ICU more often than EP patients (82.0% vs. 52.8%). LEP was not associated with delayed palliative care consultations. LEP patients more often received mechanical ventilation or ECMO than EP patients (82.2% vs. 61.8%), but time spent on mechanical ventilation or ECMO did not differ. LEP was associated with a longer hospital LOS (mean difference 4.12 days; 95% CI 1.72–6.53). However, LEP was not associated with ICU LOS.
[66]	peri-operative LOS	cross-sectional	2018	574	NA	Academic medical center in Boston	using interpreting services	Spanish, Portuguese, Chinese, Arabic, and Other	In unadjusted analyses, the median LOS decreased with increased number of interpreting events per day. Patients in Quartile 4, who had 3+ interpreting events per day, had a median LOS of 1 day. Patients in Quartile 1, who had less than one interpreting event per day, had a median LOS of 11 days. There was an association between greater frequency of interpreting events and shorter surgical patient’s peri-operative LOS.
[7]	instances of unintended harm to the patients not relating to their disease or condition	prospective cohort	2005	1083	251	Six Joint Commission accredited hospitals in the USA	Non-English speaking	NA	49.1% of reported adverse events (defined as any unintended harm to the patient not due to their underlying disease or condition) in LEP patients caused physical harm. A greater proportion of LEP patient adverse events resulted in a higher level of harm. LEP patients experienced more adverse events due to communication failure when compared to EP patients (52.4% vs. 35.9%). LEP patients experienced more adverse effects due to practitioner factors than EP patients (21.9% vs. 17.2%).
[51]	interpersonal processes of care (IPC) and cesarean delivery	cross-sectional study	2004–2006	1308	NA	Kaiser Permanente Medical Center and San	Poor or no English proficient based on interview	Spanish	At KP-MC, women who reported good or fluent English proficiency were more likely to deliver via cesarean than women with poor or no English proficiency (OR = 0.04, 95% CI 0.005–0.33). However, at SFGH, women with poor or no English proficiency were more likely to delivery via cesarean (OR = 1.61, 95% CI 0.86–3.05).
[52]	Inpatient mortality rates & obstetric trauma	cross-sectional	2009	3,211,457	545,762	Community, non- rehabilitative hospitals in California	Patient’s self-reported principal language	Spanish & Asian- Pacific Islander languages (Chinese, Japanese,	The risk-adjusted inpatient mortality for congestive heart failure, strokes, and pneumonia among Spanish and API language speakers were similar to or somewhat lower than that of EP patients. Age-adjusted rates of obstetric trauma were lower among Spanish speakers and higher among API language-speakers when compared to EP patients.
[74]	quality of acute pain treatment for obstetric and gynecological care patients	cross-sectional	2003 & 2006	185	NA	two teaching hospitals	patients who reported a need for interpreter service	Spanish	The group who responded as “Not Always” receiving interpreter services reported significantly lower scores for pain control (OR = 0.4, 95% CI 0.2–0.8), timely response (OR = 0.4, 95% CI 0.2–0.8), and perceived helpfulness from staff to respond to pain (OR = 0.3, 95% CI 0.2–0.7) than those who reported “Always” using interpreter services. Language barriers were reported by 13% of patients in the “Not Always” group as an obstacle to obtaining pain medication compared to the 8% in the “Always” group.
[71]	understanding discharge instructions	Cross-sectional	2005–2008	308	203	Urban public hospital’s general medical-surgical floor	Asking patients “How well do you speak English? and “In what language do you prefer to receive medical care?”	Spanish & Chinese	LEP participants had fewer discharge medications than EP participants (3.6 vs. 4.6). LEP patients were less likely than EP patients to have post-discharge ED visits or re-hospitalization (9% vs. 27%). Models were adjusted for clinical site, data collection time-period, and discharge time. LEP status was associated with lower odds of understanding medication category (OR = 0.63) and the outcome of medication category and purpose (OR = 0.89). LEP patients who reported language concordant discharge instructions had lower odds of understanding than EP patients (OR = 0.39).
[53]	hospital costs, LOS, 30-day readmission, and 30-day mortality risk.	observational cohort	2001–2003	5877	1146	General Medicine Service at the University of California, San	language codes collected from patient registration databases	Chinese (Cantonese or Mandarin), Spanish, Russian	Spanish and Russian-speaking patients had lower 30-day readmission rates (2.5% and 6.4%, respectively) than the EP group and the Chinese-speaking group. Chinese-speaking patients had the highest 30-day mortality (OR = 1.0, 95% CI 0.8–1.4). LEP patients had a higher odds of readmission at 30-days post-discharge than the EP group (OR = 1.3; 95% CI 1.0–1.7).
[64]	30-day readmission, LOS, & hospital expenditures	natural experiment	2007–2010	8077	1963	Academic medical center	Patient’s primary language entered at registration	Chinese, Russian, Spanish, other Asian language, and Other	LEP patients all received the intervention (Bedside Interpreter Intervention). The odds of 30-day readmission for the LEP group compared to the EP group was lower during the intervention period (0.64; 95% CI 0.43–0.95) than it was during the pre- & post-intervention periods (1.07; 95% CI 0.85–1.35 & 1.09, 95% CI 0.80–1.48 respectively).
[54]	advance care planning discussions prevalence	cross-sectional	2005–2008	369	232	medical and surgical wards of two large urban hospitals in the	If patients answered “not at all”, “not well” to the question “How well do you speak English?”	Spanish & Chinese	Participants’ English proficiency was not associated with report of advance care planning discussions.
[73]	hospital discharge	prospective cohort	2012–2013	94	79	cardiovascular, general surgery and orthopedic surgery floors in	Speaking English not at all or not well	Spanish, Cantonese, & Mandarin	Pre-post discharge preparedness and patient-reported knowledge of follow-up appointments, discharge medication administration and side effects did not differ significantly after the implementation of the bedside phone interpreters. However, in bivariate models, knowledge of medication purpose increased significantly from before compared to after the implementation (88% vs. 97%).
[77]	informed consent	prospective cohort	2012 & 2013	152	NA	Academic medical center	Hospital identification algorithm	Spanish, Cantonese, Mandarin	Researchers evaluated the impact of a bedside interpreter phone system intervention on informed consent. More patients in the post-intervention group significantly met the criteria for adequately informed consent when compared to the pre-implementation group (54% vs. 29%, respectively). Post-intervention LEP patients had statistically higher odds of informed consent in adjusted models when compared to LEP patients in the pre-implementation group (aOR = 2.56, 95% CI 1.15–5.72). The post-implementation group had statistically significant higher odds of understanding the reason for their surgery or procedure (aOR = 3.60, 95% CI 1.52–8.56). The post-intervention group also had statistically higher odds of having all their questions answered (aOR = 14.1, 95% CI 1.43–139.0). When compared to English-speaking patients, post-intervention LEP patients had 62% lower odds of adequately informed consent compared to English-speaking patients (aOR = 0.38; 95% CI 0.16–0.91).
[65]	hospital LOS and 30- day readmission rates	retrospective cohort	2004–2007	3071	NA	tertiary care, university hospital	Patients’ preferred language	Spanish, Portuguese, Vietnamese, Albanian, Russian, and Other	Patients who did not have an interpreter present on both admission and discharge days were in the hospital about 1.5 days longer than patients who had interpreters on both days. Patients who received interpreters on both admission and discharge days had a mean LOS of 2.57, compared to patients who received interpretation neither on admission nor discharge days had a mean adjusted LOS of 5.06 days. 103/423 (24.3%) patient admissions who did not have an interpreter present either at admission and discharge were readmitted within 30 days, compared to 163/963 (16.9%) of patients with an interpreter at admission only, 85/482 (17.6%) of those with an interpreter at discharge only, and 178/1192 (14.9%) with an interpreter at both admission and discharge day.
[72]	Post-discharge reported issues	retrospective cohort	2018–2019	12,294	1566	academic medical center	EHR listed a preferred language for healthcare other than English if the patient self-identified as needing an interpreter	Spanish, Cantonese, Russian, Mandarin, Vietnamese, other	More LEP patients needed assistance getting prescriptions filled (adjusted, 8.3% vs. 5.5%) and had concerns about their medications (adjusted 12.9% vs. 10.6%). While LEP patients had more post-discharge issues, there was no significant difference in issue severity.
[63]	Total joint arthroplasty (TJA)	retrospective cohort	2015–2019	4721	378	urban medical center	language preference other than English & request for interpreter services	Spanish, Chinese, other non- English language	In univariate analyses, patients with LEP who underwent TJA had longer LOS (median [IQR], 3 [2,3,4] days vs. 2 [1,2,3] days), higher costs of hospitalization (median [IQR] $15,000 [$13,000-$22,000] vs. $14,000 [$12,000-$19,000]), and were more likely to be discharged to a skilled care facility (161 patients [42.6%] vs. 889 patients [20.5%]) compared with patients with EP. There was no difference in 30-day readmission rates by language status.
[59]	unplanned ED revisit within 72 hours of discharge	retrospective cohort study	2012	32,857	2943	Mount Sinai Hospital, a tertiary medical center in NYC	used EHR patient language preference	NA	The unadjusted odds ratio between LEP status and hospital admission was 1.20 (95% CI 1.11–1.30), but the association disappeared when controlling for confounding variables. LEP patients had an OR of 1.19 (95% CI 1.02, 1.48) in unadjusted association with unplanned ED revisits within 72 hours. This association became stronger in adjusted variables with an OR of 1.24 (95% CI 1.02, 1.53).
[49]	emergency department visits and hospital admissions	retrospective cohort	2012	3784	1892	large primary health care network in Minnesota	Language spoken by patients and interpreter status in HER	Somali, Spanish, Vietnamese, Khmer, Arabic, and Other	There were significantly more total ED visits (841 vs. 620) and hospitalizations (408 vs. 343) for IS (interpreter service) patients compared with non-IS patients. The proportion of patients with at least 1 ED visit (23.7% vs. 15.4%) and at least 1 hospitalization (15.1% vs. 10.6%) was significantly higher among IS patients. Nearly twice as many IS patients had 3+ ED visits and hospitalizations than non-IS patients.
[60]	admission for emergency surgery from the ED.	retrospective cohort	2019	85,899	9874	quaternary care, urban, academic medical center	patients used hospital interpreter services	NA	LEP individuals had significantly higher odds of admission for surgery compared to EP individuals (OR 1.33, CI 1.17, 1.50), but this difference disappeared after adjusting the models. LEP Hispanics were more likely to be admitted for surgery than non-LEP Hispanics (OR 1.63, CI 1.08, 2.47).
[55]	informed consent documentation	retrospective cohort (matched chart review)	2004–2006	148	74	Public teaching hospital in San Francisco	primary language from HER	Spanish, Cantonese, and Mandarin	EP patients were more likely to have full documentation of informed consent (53%) than LEP patients, who also had evidence of interpretation (28%). Only 41% of LEP patients had a consent form in their language or had one signed by an interpreter. In the multivariate, adjusted analysis, there were no differences in documentation between the EP and LEP groups, nor between the Spanish and Chinese-speaking patients.
[56]	risk of emergency department visit admission	retrospective cohort	2017	9,641,689	1,421,385	California hospitals	Selected a non-English language as the principal language to communicate with the healthcare provider	Spanish, Mandarin, Cantonese, Tagalog, Vietnamese, and Other	LEP patients were less likely to be admitted for diabetes with short-term complications than EP patients (54.0% vs. 70.9%). More LEP patients were admitted to the hospitals than EP patients (median different of 1.3%, IQR = −1.1–5.1%).LEP patients were more likely to be admitted for COPD or asthma in older adults across all models (36.8%, 95% CI 35.0–38.6%] vs. 33.3% in EP patients (95% CI 31.7–34.9%). Admission rates for those who spoke Mandarin/Cantonese, Vietnamese, and or other had a significant difference in admission rate compared to English.
[57]	differences in discharge opioid prescribing for trauma patients	cross-sectional study	2018	1419	237	Zuckerberg San Francisco General Hospital and Trauma	English was not among patient self-reported languages spoken.	NA	41% of LEP patients were discharged on opioid medications. In multivariable models, EP patients had 1.63 adjusted increased odds of receiving any opioid prescription at discharge. EP patients received 147 oral morphine equivalents (OMEs) on average, compared with 94 OMEs for LEP patients.
[58]	birth outcomes (Cesarean sections, VBACs)	cross-sectional	2012	11,419	1149	HI hospitals that collected language preference	Preferred language noted at intake	Micronesian, Japanese, Tagalong, Spanish, Ilocan, Visayan, Mandarin, Cantonese, Chuukese, Marshallese, Tongan, Somoan, Hawaiian	There was a significant difference between primary Caesarean deliveries between EP and LEP (RR = 1.18), with a higher relative risk for patients with diabetes (RR = 1.30). There is also a significant difference in vaginal birth after Cesarean (VBAC) between EP and LEP (RR = 1.02).
[67]	LOS	prospective cohort	2011	245	124	Level 1 trauma academic emergency department	Preferred primary language recorded during registration	Spanish, Navajo, Vietnamese, Chinese, Arabic	There were no differences in mean LOS from arrival time to the time seen by a provider when comparing EP patients to LEP patients. There were also no mean LOS differences from arrival time to discharge or admission decision when comparing LEP to EP patients.
[70]	diagnostic test orders with chest & abdominal pain	prospective cohort	1997–1998	324	172	Public hospital emergency department	English speaking proficiency	Spanish, Cantonese, Hindi, Mien, Arabic, Russian, Mandarin, Korean, and other	No diagnostic test was found to be statistically significantly different between EP & LEP patients with chest pain. The frequency of ordering of CBC counts, serum electrolyte determinations, urinalyses, ECGs, and abdominal CT scans was found to be statistically different between English-speaking and non–English-speaking patients with abdominal pain.
[61]	emergency medical services scene and transport times	retrospective case- control study	2012	201	100	Albuquerque Ambulance Service and emergency	Inability to sign the EMS run report secondary to language barrier	NA	LEP patients had greater odds of calling 911 for trauma (OR, 2.5; CI, 1.4–1.5). LEP patients had longer transport times (mean difference of 2.2 minutes, CI, 0.04–4.0). LEP patients were more likely to have an electrocardiogram (ECG) done in EMS (OR, 3.7; CI 1.7–8.1) and ED care (OR = 2.0: CI, 1.1–1.3). LEP patients were more likely to leave without being seen or leave against medical advice (OR = 0.2; CI 0.1–0.7).

NA means not available in the published manuscript. Abbreviations used in the table include the following: LEP, limited English proficiency; EP, English proficient EHR, electronic health record; LOS, length of stay; ED, Emergency Department; EMS, Emergency Medical Services.

### 3.3. Screening Studies 

There were 25 studies looking at screening outcomes that met our inclusion criteria [40,79,80,81,82,83,84,85,86,87,88,89,90,91,92,93,94,95,96,97,98,99,100,101,102]. The most common screening type among these studies was cancer screening, including cervical, breast, and colorectal cancers. Other screening topics included HIV testing, Hepatitis B testing, and cholesterol screening. The majority of these studies were cross-sectional, with three retrospective cohort studies [40,79,95], and one randomized controlled trial [88]. Datasets that were used to conduct some of these studies include the California Health Interview Survey (CHIS), the Behavioral Risk Factor Surveillance System (BRFSS), the Texas Behavioral Risk Factor Surveillance Survey, and the Medical Expenditures Panel Survey (MEPS). One study used data from the Study of Women’s Health Across the Nation (SWAN) to conduct their research [87]. Two studies looked at the use of interpreter services and their relationship with breast and cervical cancer screening use [83,102]. The data used for screening studies are generally older, with the earliest being from 1995. Most studies in this group used data from before 2015, with the most recent data being from 2018. Within the studies that specified language groups, nine studies looked at Spanish speakers, six studies looked at Chinese languages (Mandarin and Cantonese), nine studies looked at other Asian languages (including Korean, Vietnamese, Cambodian, Laotian, Japanese, Thai, Tongan, and Khmer), and three studies looked at Russian, Arabic, Somali, and Amharic. As in the other healthcare categories, individuals who spoke Spanish and Asian languages were the populations most often included in the studies. A majority of these studies were ranked as low or moderate in terms of quality. This was mainly due to their cross-sectional study design, which limits the ability to determine causality, small LEP sample sizes, differences in LEP definitions, and limited language groups included, which can limit the generalizability of these studies.

Overall, the screening studies showed that LEP individuals were less likely to receive cervical, breast, and colorectal cancer screening compared to EP individuals. However, there was some variability in these findings. The impact of LEP on breast cancer screening was the most variable. There were four studies that showed no significant difference between LEP and EP populations in mammogram screenings [40,80,97,101]. However, five studies showed that LEP individuals had lower odds of receiving a mammogram compared to EP individuals [83,85,87,90,100]. In a study by Sheppard et al., endorsement of breast cancer screening was more likely among women whose primary language was English compared to those who spoke a non-English language [99]. LEP individuals were shown to be less likely to have heard of [81] and received a clinical breast exam [81,83,85,87] or to have given themselves a breast self-exam [86]. 

Cervical cancer screening studies looked at pap smears and largely found that LEP populations had lower rates of pap smear exams compared to EP populations. Eight studies found that LEP individuals were significantly less likely to have had a pap smear regularly or ever compared to EP individuals [81,83,85,87,88,90,91,101]. There were four studies that found no statistically significant impact of LEP on cervical cancer screening among Chinese- [97] and Spanish-speaking populations [40,80,102]. 

Of the studies that examined colorectal cancer screening (CRC), five found that LEP respondents were less likely to utilize these screening services [89,92,95,97,101]. One study found that LEP Latino men were the least likely to report CRC test use compared to non-LEP Latino men, non-LEP and LEP Latino women, and all non-Latino subgroups [84]. Another study found that, compared to non-LEP Mexican-Americans, those with LEP were less likely to have had CRC testing [89]. A majority of the studies that analyzed CRC screening looked at tools such as the fecal occult blood test, endoscopy, or colonoscopy. One study looked at CRC screening using a multi-target DNA (mt-sDNA) stool test and found that LEP patients were less likely to successfully complete the mt-sDNA test compared to EP patients. In this study, the return times for completed tests among LEP patients were twice as long as the return times of EP patients [79]. There were two studies that showed no impact or a mixed impact of LEP on CRC screening. Breen, Rao, and Meissner found no significant difference between LEP and EP Mexican-Americans in CRC screening [80]. A study by Jacobs et al. found that there were significantly lower rates of fecal occult blood testing among the LEP population. However, after the implementation of interpreter services, the significant differences in screenings between the LEP and EP populations disappeared [40]. 

Some studies looked at additional factors that could influence the impact of LEP on screening, such as the combination of LEP and language concordance with providers, border residence status, and low health literacy. In a study that analyzed low health literacy (LHL) and LEP both separately and together, LEP alone was not significantly associated with meeting screening guidelines (breast, cervical, and CRC). However, respondents with both LHL and LEP were less likely to meet breast cancer and colorectal cancer screening guidelines [98]. Another study reported that LEP-only respondents were less likely to meet CRC screening guidelines than LHL-only respondents [97]. Both articles examined patient-provider language concordance among the LEP population, but only one found that not having a language-concordant provider was significantly associated with lower utilization of a mammogram [98]. Another study that analyzed patient–provider language concordance found that those in the language-discordant cohort (did not speak English at home and no one at their providers spoke their language) were just as likely as the English-concordant cohort (spoke English at home) to be adherent to CRC screening guidelines [95]. The last of the four studies found that Spanish-speaking Texan Mexican-Americans who were border residents had low rates of breast and cervical screening use compared to those who were Spanish-speaking and non-border residents. However, after controlling for enabling factors (health insurance, income, and a usual source of care), the significance of the language of the interview and border residence disappeared among participants [85]. Studies like these demonstrate that it can be difficult to isolate the effects of LEP from interrelated socio-economic, geographic, and language service provision in the healthcare setting. 

There were three studies that looked at non-cancer-related screenings. One study looking at Hepatitis B (HBV) found that LEP men who spoke Vietnamese were more likely to report past HBV testing compared to the LEP population [82]. Another study looked at HIV and found that Spanish-speaking LEP men who have sex with men were less likely to receive HIV testing compared to their EP counterparts [96]. Kenik, Jean-Jacques, and Feinglass looked at cholesterol screening and found that LEP Spanish speakers were more likely to never have been screened for high cholesterol compared to the EP population [93]. Differences in the studies may be due to differences in linguistic and cultural groups (two studies among Spanish-speaking populations and one among Vietnamese populations), as well as the type of screening. Please see a list of studies included in this category with key summary information in Table 5 below. 

**Table 5:** Screening Study Details.

Screening Studies Citations	Health Outcome	Study Design	Study Period	Total Sample Size (N=)	LEP Sample Size (n=)	Setting	LEP definition	Languages	Study Outcomes (LEP Related)
[80]	cervical, breast, and two types of colorectal cancer test use	cross-sectional	2001	9079	Men = 1786; Women = 2425	California	Respondent took the interview in Spanish	Spanish	There was no significant difference between LEP and EP among Mexican-American women who had a mammogram and pap test. There was no significant difference between LEP and EP among Mexican-American men and women who had a colorectal cancer screening test.
[81]	knowledge and utilization of breast and cervical cancer early detection practices	cross-sectional	NA	135; cervical cancer survey sample = 35	63; cervical cancer survey sample = 21	community sites in New York City	Participants assessed their English speaking ability as not at all, poor, or average	Chinese	EP women were more likely to have heard of the clinical breast exam (38%), compared with the women who judged their language abilities as either totally lacking (15%) or else poor (13%) and were more likely to have had a clinical breast exam in the previous year compared to LEP participants. EP participants were more likely to believe that they needed a pap smear compared to LEP participants (*p* < 0.01), and were more likely to have had a pap smear (50% EP v 28.6% poor English and 28.6% not at all) within a year of the survey.
[82]	serologic HBV testing	cross-sectional	2002	509	262	Seattle, Washington	English proficiency determined by “speaks fluently or well”, “speaks quite well”, and “does not speak well or at all”	Vietnamese	LEP was independently associated with higher odds of past HBV testing (OR = 2.5; CI = 1.3–4.7) compared to high English proficiency.
[83]	receipt of mammogram, clinical breast exam, and pap smear	cross-sectional	2002–2003	1708	1284	California	Preferred language to speak to doctor or medical provider	Cambodian, Laotian, Thai, and Tongan	LEP immigrants had significantly lower odds of receiving a mammogram (OR = 0.46), clinical breast exam (OR = 0.59), and pap smear (OR = 0.40) compared to EP immigrants.
[84]	colorectal cancer screening uptake	cross-sectional	2008	99,883	2362	United States	Survey was completed in Spanish	Spanish	48.2% of LEP Latino men had the lowest adjusted screening rates compared to all the other Latino subgroups, which include Latina women with LEP (56.2%). Compared to non-Latino White men, LEP Latino men were the least likely to report colorectal cancer (CRC) test use (AOR 0.47; CI 0.35–0.63).
[85]	cancer screening	cross-sectional	2000- 2004	2399	1020	Texas	Language of interview	Spanish	Women that did the interview in Spanish and are border residents are less likely to utilize screening services. Those interviewed in Spanish are associated with a lower likelihood of having a pap smear (OR = 0.732, CI = 0.537, 0.998), clinical breast exam (OR = 0.489, CI = 0.383, 0.624), mammogram within the past two years (OR = 0.660 CI = 0.442, 0.986) after controlling for age and educational differences.
[79]	colorectal cancer screening completion rates	retrospective cohort study	2015–2018	412	103	Primary care clinic in the Midwest	identified in the EHR need for an interpreter	Somali, Cambodian, Vietnamese, Arabic, and other.	The percentage of mt-sDNA tests without useful results was 53.4% (55/103) among patients with LEP compared to 29.1% (90/309) among EP patients (*p* < 0.0001). This study demonstrates a significant disparity in colorectal cancer screening completion using the mt-sDNA test among populations with LEP.
[86]	cancer screening health behaviors	cross-sectional	NA	99	NA	Community center for refugees and immigrants	Asked to rate their English speaking ability.	Russian	English language was the only acculturation measure that was significantly related to behaviors and outcomes. Women who spoke and understood English better were more likely to conduct a breast self-exam (*p* < 0.05).
[87]	receipt of Papanicolaou tests, clinical breast examinations, and mammography	cross-sectional	1996- 1997	1247	No English = 278; Another language more fluently than English = 66	Oakland, CA; Los Angeles, CA; and Newark, NJ	Asked what language they usually read and spoke	Spanish, Cantonese, or Japanese	Not speaking or reading English (Pap: OR = 0.43, CI = 0.34, 0.54; CBE: OR = 0.44, CI = 0.35, 0.57) or speaking another language more fluently than English (Pap: OR = 0.50, CI = 0.35, 0.72; CBE: OR = 0.55, CI = 0.38, 0.80) significantly reduced the likelihood of receipt of Pap testing or CBE (*p* < 0.01). Those who reported not speaking or reading English were less likely to receive a mammogram (OR = 0.63, CI = 0.50, 0.80).
[40]	receipt of preventative health screenings	retrospective cohort study	1995–1997	4380	327	Four HMOs in New England	Use of interpreter services	Spanish & Portuguese	For receipt of screening services the study found significantly lower rates of FOB testing and rectal exams, but no significant difference in mammograms, breast exams, and pap smears, in the LEP compared to the EP population. After implementation of interpretation services the significant differences in screenings between LEP and EP populations disappeared.
[88]	regular cervical cancer screening	randomized controlled trial	2003- 2004	473	NA	Washington, DC metropolitan area	asking participants their ability to read, write, listen to, and speak English, ranging from “not at all” to “very good.”	Mandarin, Cantonese, Taiwanese, and Fuzhou	Women with higher English proficiency were more likely to have received regular Pap tests than women with LEP (OR, 1.39; CI, 1.13–1.72).
[89]	colorectal cancer test rates	cross-sectional	2005	18,304	590	California	Speaks “no English”, or “does not speak it well” at home	Spanish	Those with LEP were 1.68 times more likely to have never had any CRC test (*p* < 0.01) (blood test or endoscopy). Among Mexican Americans, non-LEP respondents were significantly more likely to have had fecal occult blood test (FOBT) only (10% vs. 16%; *p* = 0.01), both tests (11% vs. 29%; *p* < 0.01), and to have ever had any test (45% vs. 67%; *p* < 0.01), compared to LEP respondents.
[90]	breast and cervical cancer screening behaviors	cross-sectional	1998- 1999	438	NA	Maryland	English language proficiency was assessed by asking respondents to rate their English	Korean	Korean women who speak some English (OR = 1.98; CI, 1.07, 3.67) and those who speak English very well (OR = 2.41; CI, 1.03, 5.62) reported greater odds of having a mammogram compared to those that speak little English.
[91]	regular cervical cancer screening	cross-sectional	2000	459	NA	Maryland	Spoken English proficiency ranked as none/little; average, good/fluently	Korean	In the bivariate analysis, spoken English proficiency was identified to be significantly related to having regular pap smears (*p* < 0.05).
[92]	cancer screening	cross-sectional	2000–2001	55,428	NA	California	Does not speak English at home	Spanish, Mandarin, Cantonese, Vietnamese, Korean, or Khmer	Individuals who do not speak English at home were less likely to get screened for colorectal cancer (OR 0.75; CI, 0.58–0.98).
[93]	cholesterol screening	cross-sectional	2011	389,039	24,509	United States	Questionnaire completed in Spanish	Spanish	There was a significant difference between LEP (68.8%) and EP (88.7%) population in cholesterol screening within the past 5 years (*p* < 0.000). LEP Spanish speaking individuals were more likely to never have been screened for cholesterol (OR = 1.43; CI, 1.22–1.69) compared to EP individuals even after controlling for socio-demographic factors.
[94]	cervical cancer screening behaviors	cross-sectional	2015	97	43	a Midwestern city	Spoke English not at all or not too well	NA	23.1% of LEP individuals who spoke English not at all had ever received a pap smear. 56.7% of LEP individuals who spoke English not too well had ever received a pap smear.
[95]	colorectal cancer screening rates	retrospective cohort study	2002–2006	23,297	1703	NA	Not comfortable conversing in English	Spanish	Non-English speakers had a lower use of colorectal cancer screening (30.7% vs. 50.8%; OR, 0.63; CI, 0.51–0.76). The adjusted odds of being current with CRC screening was lower for those in the Other Language-Concordant cohort compared to those in the English-Concordant cohort (OR, 0.57; CI, 0.46–0.71). The Other Language-Discordant cohort did not statistically differ from the English-Concordant cohort (OR, 0.84; CI, 0.58–1.21).
[96]	HIV testing	cross-sectional	2012–2015	304	194	North Carolina	Speaking comfortably in only Spanish	Spanish	LEP men who have sex with other men were 0.31 times less likely to receive HIV testing compared to EP (CI, 0.16–0.57).
[97]	meeting colorectal cancer screening guidelines	cross-sectional	2007	15,888	539	California	LEP is defined as self- reporting speaking English “not well” and “not at all.”	Mandarin, Cantonese, Korean, and Vietnamese	Individuals with LEP only (OR = 0.60) and LEP plus limited health literacy (OR = 0.52) were significantly less likely to meet colorectal cancer screening guidelines. Among the 539 individuals in the sample with LEP, 54.5% had a language- concordant provider.
[98]	meeting guidelines for cervical, colorectal and breast cancer screening	cross-sectional	2007	cervical= 632; colorectal = 488; breast = 326.	cervical = 201; colorectal = 181; breast = 153	California	self-reporting speaking English “not well” and “not at all”	Cantonese and Mandarin	LEP was not independently significantly associated with meeting any of the screening guidelines for breast, cervical, or colorectal cancer comparing LEP to EP among the Chinese population.
[99]	endorsement of breast cancer screening	cross-sectional	NA	200	91	Washington, DC	Primary language labeled as “other”	Amharic and other	Endorsement of breast cancer screening was more likely among women whose primary language was English compared to those who spoke a non-English language (OR = 3.83; CI: 1.24 to 11.87).
[100]	colorectal, breast, and cervical cancer screening	cross-sectional	2012- 2013	NA	NA	Northern California outpatient healthcare system	Primary language is not English	NA	LEP individuals are 0.81 times less likely to receive a mammography screening (CI: 0.71, 0.92). LEP individuals are 0.79 times less likely to receive a colorectal cancer screening (CI: 0.72, 0.87).
[101]	accessing coloscopy, mammography, and papanicolaou smear screening.	cross-sectional	2013- 2015	1298	NA	New York City	NA	NA	English language proficiency was a significant barrier for some screening methods such as colorectal cancer screening with colonoscopy, and cervical cancer with pap smear, but not for mammography. Non-English speakers are significantly less likely to have a pap smear (OR = 0.24, CI= 0.14–0.41) compared to English speaking participants.
[102]	papanicolaou smear screening access	cross-sectional	2007–2008	318	271	Boston, MA	Need a translator during a healthcare encounter.	Spanish	There was no significant difference in likelihood of having less than 5 or 5 or more lifetime pap smears between women who report that they need a translator during a healthcare encounter.

NA means not available in the published manuscript. Abbreviations used in the table include the following: LEP, limited English proficiency; EP, English proficient; HER, electronic health record.

### 3.4. Specific Condition Studies

We found 40 studies investigating specific conditions that met our inclusion criteria [12,13,103,104,105,106,107,108,109,110,111,112,113,114,115,116,117,118,119,120,121,122,123,124,125,126,127,128,129,130,131,132,133,134,135,136,137,138]. Overall, these studies showed many similar patterns to the other studies in our analysis, such as small LEP sample sizes and wide variability in how the LEP population was defined. Spanish was overwhelmingly the most studied language in this group, with 33 studies explicitly including Spanish speakers. Other languages studied seemed to vary regionally, with Chinese languages being the second most studied. As was the case throughout our review, nearly all of the studies in this category were observational, and half of them (20 in total) were retrospective chart reviews. This choice of study design makes practical sense, as intervention studies of specific conditions (such as cancer drug trials, for example) are ethically complex and require significant institutional resources. Our analysis found two papers using data from one randomized controlled trial [123,139]. This trial was seeking to compare two different methods of language interpretation. There were no other interventional studies in this category. The types of conditions studied skewed toward chronic conditions, including diabetes and related conditions (13 studies), treatment of various cancers (four studies), mental health conditions (five studies), hypertension (three studies), and asthma (two studies). Two studies looked at Hepatitis B serologic status. One study investigated TBI, one investigated heart failure, one looked at blood clot prevention, and one looked at acute stroke care. The remaining seven studies investigated outcomes for different types of surgical procedures, including cataract surgery and post-tonsillectomy hemorrhage. Studies primarily looking at more acute cancer-related surgery outcomes (two studies) are grouped with other surgical studies due to similarities in the types of health outcomes being investigated. 

Because of the wide variety of clinical targets for specific conditions, it is more difficult to draw general conclusions about outcomes in this category of study. However, the vast majority of studies in this category investigated conditions, whether acute or chronic, that have at least one agreed-upon, objectively measurable, condition-specific benchmark. In conditions such as diabetes, for example, the laboratory measurement of HgbA1c is a benchmark; in hypertension, the benchmark is a vital sign; in acute stroke care, the benchmarks include door-to-imaging time and administration of tPA; in neurologic conditions such as dementia and TBI, the benchmarks are the results of standardized clinician-administered cognitive testing, etcetera. In studies looking at objectively measurable clinical outcomes such as these, outcomes were almost universally worse for LEP populations. There were a few interesting exceptions, however. Four studies appeared to find no significant differences among study populations; three of these were looking at diabetes and one at acute stroke. However, one of those studies looked only at LEP patients in order to compare different types of language interpretation and found poor glycemic control across the entire sample [139]. A second study in this group, also looking at diabetes, found poor glycemic control across all Hispanic patients, regardless of preferred language [122]. The third study of diabetes that appeared to find no significant difference in outcomes for LEP populations was actually looking at a group of clinics that had a high proportion of bilingual staff, and although the data analysis was otherwise rigorous, this study did not account for the potential interfering factor of language concordance between staff and LEP patients [124]. Of the four exceptions, the study of acute stroke is the most interesting, finding no significant difference in outcomes for LEP patients in any of the acute stroke care benchmarks, but it is limited in that it looked exclusively at a single stroke center and had a relatively small sample size [104].

In some studies, particularly those looking at hospital-based treatments such as surgeries or acute treatment of heart failure [132], target health outcomes were more uniformly defined and similar to the findings outlined in the other hospital care studies we found. Such studies tended to look at outcomes such as the rate of any complication, procedure-related mortality, hospital length of stay, 30-day readmission rate, and discharge disposition. This group of studies had the highest proportion of neutral outcomes; that is, five of the seven studies in this group found no significant difference in outcomes for LEP populations. It should be noted, however, that all of the studies in this group had relatively small LEP patient sample sizes, and most were looking at only a single hospital or care center. 

A third group of studies looked at outcomes that suggested whether or not recommended care had taken place. Included in this group are two studies of surgeries that looked at whether or not a recommended procedure took place and a study of Serious Mental Illness (SMI) that looked at contact points for mental health care [116]. The surgery studies were looking at cataract surgery [106] and at various types of surgery for breast cancer [125]. The outcomes of these studies are more challenging to interpret. In the cataract study, when those with visually significant cataracts were compared with those who had obtained cataract surgery, those who spoke English were nearly twice as likely to have obtained surgery [106]. However, this study was a cross-sectional population survey, meaning that cause and effect cannot be inferred. In the breast cancer surgery study, rates of recommended procedures were examined across many types of cancer, and while it seems encouraging that no significant difference was found between groups, the study was limited to a single center, and the LEP population size was less than 60 people [125]. The most interesting results were from the SMI study, which was more of an attempt to gather information about how LEP patients with SMI first contacted mental health services in a single urban area. This study found that LEP patients tend to prefer outpatient contact over emergency department contact. However, this study included data from a large outpatient clinic that was specially designed to serve exclusively East Asian LEP mental health patients, with a large number of multilingual staff, and the study authors themselves note that this could have biased the results [116]. 

Another group of studies looked at patient adherence to best practices for self-management of specific conditions, including three looking at diabetes self-management [111,119,128], one looking at treatment adherence for cardiovascular disease [136], and one looking at adherence to warfarin in the treatment of blood clots [133]. One study showed that LEP Latinos were less likely to adhere to oral medications and insulin compared to EP diabetic patients [111]. A second study showed that LEP Latinos were more likely to have a less-than-daily practice of self-monitoring blood glucose among Type 2 diabetic patients treated pharmacologically, although there was no significant difference shown among Type I diabetics [119]. These mixed results for diabetes management adherence may be due to differences among language groups and differences in measures of self-management. In the study by Njeru et al., while LEP patients had a lower percent likelihood of meeting recommendations for A1c and LDL levels when adjusted for sociodemographic risk factors, there was found to be no significant association between LEP and diabetes management. The one exception was blood pressure, where LEP individuals were more likely to meet the guidelines than non-LEP patients [128]. Both the warfarin and cardiovascular disease studies showed a lower likelihood of LEP populations being in the therapeutic range (for warfarin dosing) and having good adherence to cardiovascular disease treatment medications, including lipid, blood pressure, and glucose medications. 

The remaining studies in this category targeted depressive disorders. Because of the nature of this condition, all three of these studies tracked outcomes using a subjective symptom scale that relied on patient self-report. Interestingly, each of the three depression studies used a different symptom scale, making cross-study comparisons impossible. However, the fact that so few studies of this type of mental health condition appeared in our review is notable and suggests the additional complexity involved in collecting subjective or qualitative health data in LEP populations. Two of these studies appeared to find a link between better English proficiency and an increased likelihood of either obtaining a depression diagnosis [123] or testing positive on a depression screening test [127]. Interestingly, both of these studies were focused on Asian patients. In the third study, which focused on Mexican-American patients exclusively, the depression symptom scores of the LEP group were found to increase at a faster rate over time [121]. Overall, these studies again bear out the overall pattern of poorer health outcomes in LEP populations. Please see a list of studies included in this category with key summary information in Table 6 below.

**Table 6:** Specific Condition Study Details.

Specific Condition Citations	Health Outcome	Study Design	Study Period	Total Sample Size (N =)	LEP Sample Size (n = )	Setting	LEP Definition	Languages	Study Outcomes (LEP Related)
[103]	clinical diagnosis of diabetic peripheral neuropathy recorded in the EHR	cross-sectional	2003–2008	12,681	1626	Kaiser Permanente of Northern California	asked if respondents had difficulty understanding English	NA	LEP was independently associated with absence of clinical documentation of diabetic peripheral neuropathy in the EHR, despite reporting symptoms when surveyed. [RR 0.80 (0.68, 0.94)]
[104]	acute stroke care benchmarks and mortality rate	retrospective cohort	2013–2016	928	282	UC Irving Stroke Center (inpatient)	preferred language as indicated on admission	Spanish, Other	There was no statistically significant difference in acute stroke care benchmarks between LEP patients and patients whose preferred language was English
[105]	access to specialist care for colorectal cancer	cross-sectional	1999–2000	1079	75	Participants from 9 northern California counties	self-report of language spoken at home	Spanish	White LEP people reported significantly more problems with access to care than other groups, including Hispanic and Asian LEP people (*p*< 0.001).
[106]	cataract surgery	cross-sectional	1997–1999	4774	NA	outpatient clinics in Arizona, specifically Pima and Santa Cruz	Preferred language on interview was “mostly Spanish” rather than “Spanish and English”	Spanish and English	Comparing those who obtained cataract surgery with those having visually significant cataract (i.e., those needing surgery), speaking English (OR, 1.80; *p* = 0.04) was significantly associated with having obtained cataract surgery, even after adjusting for demographic variables and other potential risk factors.
[107]	participation in diabetes self-care measures	cross-sectional	2009–2010	250	250	outpatient clinics in rural California	not stated—study included only Spanish- speaking participants	Spanish	Spanish-speaking type 2 diabetes patients who had a Spanish-speaking provider reported engaging in diabetic foot care more frequently than those who did not have a Spanish-speaking provider (1.4 days vs. 0.7 days per week, *p*= 0.01).
[108]	Clinical variables related to diagnosis and treatment of squamous cell carcinoma	retrospective cohort	2014–2019	477	51	single cancer treatment center in Boston	preferred language at time of patient registration	Spanish, Mandarin, Vietnamese, Farsi, Greek and Haitian Creole	The LEP patients were diagnosed with cancer at a later overall stage (*p* = 0.03) and less frequently treated with surgery alone compared to English speaking patients (*p* < 0.001). After adjusting for stage and site, LEP patients were significantly more likely to receive primary surgical management compared to primary non-surgical management [OR =2.29 95% CI (0.93, 5.58), *p* = 0.008].
[109]	high BP measurement in the absence of a self-reported diagnosis of hypertension, and/or a hypertension med prescription	cross-sectional	1993–1994	2597	NA	Community setting, 5 southwestern states	Used three separate but overlapping LEP definitions, and reported on each separately:	Spanish	Those who used Spanish more than English for mass media were twice as likely to have undiagnosed hypertension than those who used primarily English.
[110]	Cancer-related surgery outcomes, including LOS, 30-day ED revisit, all-cause	retrospective cohort	2012–2017	2467	824	Inpatient; single urban hospital	LEP status was determined by examining language concordance between	NA	After adjusting the results for insurance status, comorbidities, and other factors, there was no difference in surgery outcomes found between the LEP and EP groups
[111]	adherence to prescribed hypoglycemic medication	retrospective cohort	2006–2012	30,838	3205	Kaiser Permanente Northern California	preferred language was Spanish in electronic health record	Spanish	LEP Latinos were more likely to be non-adherent to oral medications and insulin than English-speaking Latinos [RRs 1.11–1.17, *p* < 0.05] or Whites [RRs 1.36–1.49, *p* < 0.05].
[113]	HgbA1c	cross-sectional	2005–2006	6738	510	Kaiser Permanente Northern California	self-report; DISTANCE survey asked if respondents had difficulty understanding	Spanish	Among LEP Latinos, having a language discordant physician was associated with significantly poorer glycemic control (OR 1.98; CI 1.03–3.80).
[112]	LDL and systolic BP	retrospective cohort	2005–2006	7359	542	Kaiser Permanente Northern California	self-report; DISTANCE survey asked if respondents had difficulty understanding	Spanish	There were no statistically significant differences between LEP and non-LEP patients in terms of BP control. Among Latinos, LEP patients were less likely to have poor lipid control than English-speaking patients (odds ratio, 0.71; 95% CI, 0.54–0.93), with no difference by LEP patient–physician language concordance. LDL control was poor across the entire study group.
[114]	Undiagnosed dementia	cross-sectional	2011	7385	362	Used data from a nationally representative study	Responding “not well” or “not at all” when asked how well patients understand or speak	NA	Older adults with LEP were found to have 3.10 higher odds of possible dementia (95% CI 2.06–4.66). LEP was associated with significantly greater odds of undiagnosed dementia (OR = 2.95, 95% CI 1.70–5.12). LEP accounted for 87.6% of the foreign-born status effect on possible dementia, ad it explained 56.1% of the foreign-born status effect on undiagnosed dementia.
[115]	adherent to antipsychotic medications, hospitalization and health care costs	retrospective cohort	1999–2004	31,560	2823	San Diego County	self-reported preferred language	Spanish and Asian languages	A greater proportion of LEP Latinos were adherent compared to English proficient Latinos (41% vs. 36%, respectively, *p* = *0*.002). A lower proportion of LEP Asians were adherent compared to their English proficient counterparts (40% vs. 45%, respectively, *p* = 0.034). LEP Latinos were less likely than English proficient Latinos to experience psychiatric admissions (17% vs. 21%, *p* < 0.001); non-psychiatric admissions (20% vs. 22%, respectively, *p* = 0.014); and overall inpatient admissions (33% vs. 38%, respectively, *p* < 0.001). LEP Latinos and Asians had the lowest overall costs- healthcare services and pharmaceuticals per year (15,883 USD and 15,138 USD, respectively) compared to other groups (adjusted for adherence).
[116]	first point of contact with public mental health services, service utilization for 18	retrospective cohort	2000–2005	9243	1108	public mental health services in San Diego county; included	Preferred language as listed in EMR	Spanish, Vietnamese, Tagalog	LEP patients are significantly less likely to first contact mental health services through an emergency department, and more likely to use an outpatient clinic. They are also significantly less likely to use emergency services within the first 6 months of treatment, and more likely to seek outpatient services (*p* < 0.001 for each comparison).
[117]	HgbA1c, BP, LDL	cross-sectional	2003–2018	5017	889	national survey administered in a community setting	Anyone who completed the survey in a language other than English or used an interpreter	Spanish, Other	Compared to English-speaking participants, the LEP group that spoke a language other than Spanish (199 participants) were more likely to have elevated HbA1c (OR = 1.6, 95% CI = 1.1, 2.4) or a combination of elevated HbA1c, elevated LDL, and elevated BP (OR = 3.1; 95% CI = 1.2, 8.2).
[118]	30-day post-op complications and readmissions after non- emergent infrainguinal bypass surgery	retrospective cohort	2007–2014	261	51	Inpatient, single urban hospital	Preferred language in medical record	Spanish, Portuguese Creole, Haitian Creole, Albanian, Other	No statistically significant difference in outcomes was found between the LEP group and the EP group.
[119]	self-monitoring of blood glucose	cross-sectional	1994–1997	44,181	168	Kaiser Permanente Northern California	requested a materials in a non-English language, used a Spanish-speaking interviewer for survey, interviewer assessment	NA	The LEP population was more likely to have a less-than-daily practice of self-monitoring blood glucose (SMBG) among type 2 diabetic patients treated pharmacologically (OR 1.3, CI [1.2–1.5]), although there was no significant difference shown among Type I diabetics in SMBG practice. In a sub-group analysis there was a significant difference between Type I diabetic Hispanic LEP and EP populations in checking SMBG greater than 1 time daily, but not in greater than 3 times daily.
[120]	Hypertension as defined by systolic BP over 140 or diastolic BP over 90	retrospective cross-sectional	2003–2012	23,382	3269	used data from a national survey administered in a community	Anyone who completed the survey in a language other than English or used an interpreter	Spanish, Other	LEP was associated with an odds ratio of 1.47 (95% confidence interval: [1.07–2.03]) for having elevated BP
[121]	CES-D scores (depression screening tool)	prospective cohort, longitudinal	1993–2007	2945	1793	community setting in 5 southwestern states	self-report of speaking English “not at all” or “not too well” on study survey	Spanish	The CES-D scores of LEP patients increased at a more rapid rate over time during the study period.
[122]	HgbA1c	retrospective cohort	1997–1998	183	79	outpatient public clinics in Denver, CO	Record of spoken language in the administrative database, then confirmed by	Spanish	LEP patients had no significant difference in glycemic control. However, the study only looked at Hispanic patients, and noted glycemic control was equally poor for the entire sample, regardless of language ability.
[123]	Depressive disorder diagnosis and/or prescription of anti- depressant	RCT, nested cohort	2003–2005	782	NA	Primary care clinic at NYC hospital	Participants were asked if they preferred an interpreter. If yes, they were considered LEP,	Spanish, Chinese	Among BDI-FS positive patients, Chinese-speakers were less likely to be diagnosed with depression compared with English speakers (31% vs. 10%, *p* < 0.05).
[124]	HgbA1c, self-reported hypoglycemic events	cross-sectional	2011–2012	1053	793	outpatient community health centers in Northern	Language preference for the survey. Preference for a language other than English meant LEP	Spanish, Chinese	The study found no significant difference in measured health outcomes between LEP and non-LEP groups
[125]	Rates of specific breast cancer surgery, receipt of recommended breast cancer treatment	retrospective cohort	2008–2018	417	59	Outpatient comprehensive cancer center in an urban area	Requiring an interpreter	Spanish, other	No difference was found between the LEP group and EP group in terms of breast cancer outcomes. The LEP group had a lower all-cause mortality rate in the unadjusted analysis
[140]	LOS, discharge disposition, and 30-day readmission rate	retrospective cohort	2015–2019	2232	146	UCSF neurosurgical center	self-report of English not primary language and preference for interpreter services at	Spanish, Chinese	An association was found between LEP and longer LOS (incidence rate ratio 1.11, 95% CI 1.00–1.24), and discharge to skilled care (OR 1.76, 95% CI 1.13–2.72), which remained after adjusting for confounders. There was no difference in 30-day readmission rates by language status.
[126]	Glasgow Outcome Scale-Extended scores at 6 months post- injury, access to rehab	retrospective cross-sectional	1998–2005	476	42	urban Level 1 trauma center	Because of the nature of the injuries being treated, definition was twofold: If patient was	Spanish	LEP was associated with an odds ratio of 15.093 (95% CI [1.632–139.617]) of having a GOSE score indicative of severe disability 6 months post-injury. This was true even though no statistically significant difference was found for LEP patients in terms of either severity of initial injury, or access to rehab services.
[127]	Positive PHQ-2 screening test, indicating depression risk	cross-sectional	2013–2016	1532	519	Community setting	Self-report of speaking English “not well” or “not at all”	NA	The study did not find a consistent, statistically significant link between LEP and depression risk. However, among South Asians, increased depression risk was associated with greater English proficiency (OR = 3.9, 95% CI: 1.6–9.2)
[128]	diabetes management	retrospective cohort	2012–2013	13,456	1486	Minnesota Mayo Clinic and Hennepin County Medical	need interpreter services	NA	LEP patients were less likely to meet guideline outcome recommendations for hemoglobin A1C (66.9 vs. 73.9%; p < 0.000) and LDL-C (59.3 vs. 71.4%; *p* < 0.0001), but more likely to meet guideline outcome recommendations for blood pressure (83.3 vs. 75.9%; *p* < 0.000). In adjusted regression analyses LEP patients were more likely to meet guideline outcome recommendations for blood pressure <140/90 (OR, 2.02 CI [1.7, 2.4]) compared to non-LEP
[129]	HgbA1c, systolic BP, and LDL cholesterol	cohort	2007–2013	1605	1605	outpatient clinics in the Kaiser Permanente Northern	self-report of Spanish as primary language in EMR	Spanish	LEP patients who switched to a language concordant provider had significantly better A1c and LDL control vs. those who switched between two language discordant providers. After adjustment, the prevalence of glycemic control increased by 10% (95% CI, 2% to 17%; P = 0.01), and LDL control increased by 9% (95% CI, 1% to 17%; *p* = 0.03).
[130]	Incidence of post- tonsillectomy hemorrhage, and operative or non-	retrospective cohort	2015–2020	2466	1026	Inpatient head and neck surgery center in Boston	primary language preference in medical record	NA	There were no statistically significant differences in disposition or outcomes for LEP patients.
[131]	Treatment outcomes in head and neck cancer (HNC) patients receiving curative	retrospective cohort	2004–2010	131	20	Private, non- profit, urban academic medical center	Primary language spoken	Spanish, Portuguese, Russian, Vietnamese, Arabic, Mandarin, Haitian	English proficiency was significantly associated with an improved three-year locoregional control (LRC) among EP patients (82.2%) when compared to LEP patients (58.3%). LEP patients who received chemoradiation had inferior 3 year LRC when compared to the LEP patients who only received radiation (29.2% vs. 87.5%). LEP was determined to be a significant predictor locoregional failure (LRF), though the significance went away after adjusting for race/ethnicity.
[132]	30-day readmission rate	prospective cohort	2012	145	45	Columbia Presbyterian hospital in New York City	Preferred language on admission	Spanish	The hazard ratio for 30-day readmission for patients who did not speak English as a primary language was 2.2 (*p* = 0.052).
[133]	time in therapeutic range with warfarin	retrospective cohort	2009–2010	3770	241	Massachusetts General Hospital	self- reported speaking English less than “very well”	NA	LEP patients compared with non-LEP patients spent less time in therapeutic range (71.6% versus 74.0%, *p* = 0.01) and more time in danger range (12.9% versus 11.3%, *p* = 0.02). In adjusted analysis, LEP patients had lower time in therapeutic range compared with non-LEP patients (OR 1.5, CI [1.1, 2.2]), but were not at greater risk of spending more time the danger range.
[134]	Hep B serologic status	retrospective cohort	1997–2017	22,565	16,449	outpatient health center in New York City	Self-report of language preference in chart, LEP if other than English	Mandarin, Cantonese, Other	Overall, LEP status was associated with higher likelihood of HBV current or ever infection. In the multivariate analysis, specifically having Mandarin as a preferred language was associated with higher likelihood of Hep B current infection [OR 1.67 (CI 1.33–2.10)], or ever-infection [OR 1.93 (CI 1.69–2.21)].
[135]	Hep B serologic status	retrospective cohort	2000–2010	1234	1234	Outpatient clinic in Seattle, WA	Primary spoken language in medical record	Somali, Amharic, Khmer, Vietnamese, Tigrinya, Oromo, Chinese, Other	Only 8.9% of the sample was vaccinated. 56% were core positive, meaning they had been exposed to Hep B in their lifetime. There was a higher prevalence of exposure among speakers of Khmer and Oromo.
[136]	Treatment adherence for CVD risk factor controlling medications	retrospective cohort	2005	131,277	6712	Kaiser Permanente Northern California	self-report language preference	Spanish	Spanish-speaking patients were less likely than English speaking patients to be in good adherence (51% versus 57%, *p* < 0.001). When considered separately adherence for glucose lowering medications, lipid lowering medications, and BP lowering medications also showed a significant difference between Spanish and English speaking patients.
[137]	HgbA1c, LDL, BP	retrospective cohort	2013–2014	5460	1555	Hennepin County Medical Center (Minneapolis)	Preferred language in medical record	Spanish, Somali, Amharic, other	More LEP patients met BP targets (83 vs. 68%, *p* = 0.000) and obtained LDL targets (89 vs. 85%, *p* = 0.000), but this group also had worse LDL control (57 vs. 62%, *p* = 0.001).
[12]	Asthma symptom control (by ACQ score), and service utilization	prospective cohort	2004–2007	318	57	primary care clinics, 1 in East Harlem, NY, and 1 in New Brunswick, NJ	report 1) that English was not their native language, and 2) that they could not speak as well as a native speaker	Spanish	Hispanic LEP patients had significantly higher ACQ scores (higher scores mean worse symptom control), at both the 1 month and 3-month follow-ups, with the most striking difference at the 3-month follow-up. This finding remained significant even after participants over 65 were excluded from the sample, and remained significant in the multivariate analysis. LEP patients also had significantly more exacerbations requiring inpatient follow-up, again even when controlling for age (*p* < 0.05 for all comparisons).
[13]	Asthma symptom control (by ACQ score), and service utilization	prospective cohort	2009–2011	268	38	Outpatient clinics in NYC and Chicago	Self-report of speaking English “very poorly,” “poorly” or “fairly” on initial study interview	Spanish	Hispanic LEP patients had worse asthma control (*p* = 0.0007) and increased likelihood of inpatient visits (*p* = 0.002). The finding persisted when results were adjusted for demographics, asthma history, comorbidities, depression, and health literacy.
[138]	Short-term clinical outcomes after surgery: LOS, mortality, any complication, and disposition to rehab	cross-sectional	2009–2017	7324	554	New Jersey inpatient neurosurgery wards	primary language recorded on admission was not English	Spanish, other	The non-Spanish-speaking LEP group had increased post-operative LOS (adjusted incidence rate ratio, 1.10; *p* = 0.008) and higher odds of a complication (adjusted OR, 1.36; *p* = 0.015).

NA means not available in the published manuscript. Abbreviations used in the table include the following: LEP, limited English proficiency; EP, English proficient EHR, electronic health record; LOS, length of stay; BP, blood pressure.

### 3.5. General Health Outcomes

There were 17 studies that looked at general health status. Of these, one looked at oral health [3], ten looked at mental health [2,21,141,142,143,144,145,146,147,148], twelve looked at general health status [2,21,141,142,143,144,145,146,147,148], and four looked at physical functioning [142,144,145,147]. The majority of the studies looked at Asian-language LEP populations, including Chinese, Korean, Khmer, Vietnamese, and Tagalog, while five included Spanish speakers, one looked at Somali, and one looked at Marshallese speakers. Most studies of general health outcomes used self-reported measures, although a few used validated measures of physical and mental functioning, and all of the studies used cross-sectional data. The sample sizes are generally larger than seen in many of the other studies of LEP populations due to the use of national and state surveys, with the largest sample size of 51,048 (LEP 3715) in an analysis of the California Health Interview Survey. The studies mainly reported on outcomes in traditional immigrant-receiving states such as California, Texas, and New York, although there were two smaller surveys conducted in the Midwest [143,149]. Self-reported health questions based on a 5-item Likert scale are common in many cross-sectional surveys, and other large surveys used included the Study of Older Korean Americans, the National Latino and Asian Americans Study, and the New Immigrant Survey. The quality of eight of the studies was low due to restricted geographic regions or sub-analyses that limited sample sizes and impacted generalizability, as well as limited variables to address potential confounding factors, such as those related to socio-economic status, or in one case, language groups. 

Having LEP was significantly associated with having fair or poor health compared to the English-proficient population in almost all studies. This also held true for mental health, where individuals with LEP were more likely to report poor mental health, mental distress, and depression than their non-LEP counterparts. In one study, the unadjusted models showed higher mental health status and lower physical health status for LEP populations compared to EP populations, but when the model was adjusted for sociodemographic variables, there was no significant relationship between LEP and mental or physical health outcomes [147]. The study by Takeuchi et al. showed that LEP men were significantly more likely to have lifetime and past-year diagnoses of depression, anxiety, and other psychiatric disorders, but that there was no significant difference in mental health diagnoses between women with LEP and those who were EP [141]. Three studies looked at activity limitations and rates of disability, and all three found that rates were higher in the LEP population, although rates of disability by LEP status differed by language group [21,144,145]. While the overall impacts on both physical and mental health outcomes for the LEP population seem clear, it is important to keep in mind that these outcomes may not be uniform across demographic and linguistic groups and that the LEP population is not monolithic. Please see a list of studies included in this category with key summary information in Table 7 below.

**Table 7:** General Health Outcome Study Details.

General Health Outcomes Citations	Health Outcome	Study Design	Study Period	Total Sample Size (N =)	LEP Sample Size (n = )	Setting	LEP Definition	Languages	Study Outcomes (LEP Related)
[143]	mental health (general distress, somatic distress, and performance distress)	cross-sectional	NA	83	NA	Community centers and Buddhist temple in Midwest	English language proficiency measures on a Likert scale of 1 (very poor to 5 (excellent)	Vietnamese	Women who reported poorer English language proficiency had greater general distress and somatic distress compared to women with higher English proficiency.
[42]	Self-rated overall health status	cross-sectional	2014	342	286	California	Participants who reported less than ‘very well’ to the question—“Would you	Korean or other	LEP participants were 4.67 times more likely to rate their overall health as fair/poor compared to their EP counterparts (CI 1.25–16.40, *p*< 0.05). Data on physical activity and smoking status were not significant.
[148]	self-rated general health, mental distress, and cognitive health	cross-sectional	2017–2018	2032	1512	Los Angeles, CA; New York City, NY; Austin, TX, Honolulu, HI;	how well participants spoke English, not at all/a little)	Korean	LEP was a significant predictor in the model for self-rated health (OR = 1.99, CI = 1.37, 2.87) and mental distress (OR = 1.43, CI = 1.04, 1.96), but not for cognitive health
[145]	activity limitation, self-rating of general health, and symptoms of depression	cross-sectional	2008–2013	1301	922	FL, NY, and TX	reported that they spoke English less than very well	Korean	LEP significantly increased the odds of an activity limitation (OR = 2.72 ), fair or poor heath (OR = 2.59), and probable depression (OR = 1.73) compared to non-LEP.
[146]	self-reported mental health	cross-sectional	2002–2003	865	481	National	Language of interview	Spanish	LEP had statistically significant worse (RR = 2.12) mental health with no psychosis
[21]	physical and mental health status	cross-sectional	2007	1745	988	California	Reported English speaking ability was not well or not at all	Spanish, Korean, Mandarin, Vietnamese, and Cantonese	Of the four chronic health conditions studied, diabetes mellitus was the only condition that was significantly different across language groups for Latinos (LEP 27.2%, EP 18.6%, English Only [EO] 13.2%). Rates of chronic health conditions did not differ according to language status for Asian immigrants. Disability rates were significantly higher in Latinos and Asians with LEP than in their counterparts with EP and EO. Individuals with LEP had poorer self-rated physical and mental health compared to both EP and EO immigrants.
[150]	Self-rated overall health status	cross-sectional	2010	381	137 do not speak English; 239 prefer to have an interpreter	community coalition in Lowell, MA	Do not Speak English; Prefer to have an interpreter in healthcare settings; prefer to	Khmer	In bivariate analysis, Speak English (OR= 3.3) and prefer to receive health information in English (OR = 1.96) are more likely to have excellent, very good, or good self-reported health. Prefer to have an interpreter in healthcare settings are less likely (OR = 0.38) to have excellent, very good, or good health. In the multivariate analysis, LEP was not a significant predictor of self-reported heath after taking age, sex, and disability into account.
[149]	Self-rated overall health status	cross-sectional	NA	378	79	Diabetes Prevention Program in Arkansas and Oklahoma	individual reported speaking English not well or not at all	Marshallese	Regression analysis showed that participants who reported speaking English not at all/not well were significantly less likely to report better general health (excellent health or good health vs. fair/poor health) compared with those who reported speaking English very well (OR = 0.22, CI:.09, 0.54).
[142]	self-rated overall health status, mental health, and physical functioning	cross-sectional	2000	205	NA	New York City	Speaking English proficiency rated as Not at all, Not too well, Some what, or Very well	Chinese, Korean	Language proficiency predicated variance in physical functioning, general health, and mental health.
[147]	Physical and Mental Component Summary Medical Outcomes Study Short-Form	cross-sectional	2009–2011	439	293	Massachusetts	the Basic English Skills Test Plus (BEST Plus) low proficiency (0–329), moderate proficiency (330–598)	Somali	Having low English proficiency (β = 1.75, *p* = 0.02) were associated with higher mental health scores in the unadjusted model but had no significant relationship in the adjusted model. Low English proficiency were significantly associated with lower physical health scores in the unadjusted β = −3.33, *p* < 0.00) but not the adjusted models. No impact of moderate proficiency was found.
[144]	self-rated overall health status, mental health, and physical functioning	cross-sectional	2005	1196	NA	California	English proficiency from “only English” (1) to “not at all” (5).	NA	Lower levels of English proficiency were associated with higher odds of reporting worse General Health [OR = 1.85 (1.56, 2.19)], more Limited Physical Days [OR = 1.23 (1.02, 1.49)], and more Limited Combined (mental and Physical Health) Days [OR = 1.23 (1.03, 1.47)].
[151]	Self-rated overall health status	cross-sectional	2003–2004	763	148	National	self- rated respondents spoke English- ‘‘not well/ not at all’’	NA	Logistic regression showed that those who spoke English very well had lower odds of rating their current health as good/fair/poor than those who did not speak English well/not at all.
[2]	Self-rated overall health status and mental health	cross-sectional	2001	18,000	1242	California	reported speaking English not well or not at all	Spanish, Cantonese, Mandarin, Korean, Vietnamese, and Khmer,	In bivariate analysis LEP older adults had significantly higher proportions that reported poorer general and emotional health status than older adults who speak English only. In multivariate analysis, LEP older adults had increased risk of being in fair or poor health (RR = 1.68, *p* < *0*.001) and of feeling sad all or most of the time (RR = 2.49, *p* < *0*.001) compared with English only speakers.
[152]	Self-rated overall health status	cross-sectional	2007	51,048	3715	California	Self-reporting speaking English “not well” and “not at all.”	Spanish, Mandarin, Cantonese, Korean, and Vietnamese	LEP pop were significantly more likely to report poor health status compared to those with English proficiency (42.9% vs. 14.9%). Compared to those with English proficiency and adequate health literacy, the odds ratios of poor health were 2.10 (CI: 1.70–2.58) for LEP. LEP was also significantly associated with poorer health status vs. the reference among Latinos (OR: 2.01; CI: 1.51–2.69), Vietnamese (OR: 5.46; CI: 2.47–12.05), Whites (OR: 2.05; CI: 1.03–4.08), and Other race/ethnicity (OR: 2.05; CI: 1.34–3.12).
[3]	oral health	cross-sectional	2008	1870	231	New York City	Self- reported ability to speak English was poor	Chinese, Spanish	Among Chinese group, those who reported that their ability was fair or better were 2.24 (1.02, 4.96) more likely, (compared to Chinese spoken English poor) to have gone to a dentist in the past year. Difference among Hispanic LEP group was non-significant.
[141]	lifetime and 12-month rates of any depressive, anxiety, and substance abuse disorders	cross-sectional	2002–2003	2095	797	National study	Individual reported that spoken English was “fair/poor.”	English, Spanish, Mandarin, Cantonese, Tagalog, and Vietnamese	In the regression analysis non-LEP men were significantly less likely than LEP men to have any lifetime (OR = 0.44) or 12 month (OR = 0.29) depressive disorder, lifetime (OR = 0.51) or 12 month (OR = 0.45) anxiety disorder, or lifetime (OR = 0.52) or 12 month psychiatric disorder (OR = 0.45). There was no significant difference in mental health diagnoses between women with LEP and those who were English proficient.
[153]	Self-rated overall health status	cross-sectional	2010–2013	705	self-rated English proficiency—473; ever need medical interpreter—244	San Francisco, CA	self-rated spoken English proficiency (“not at all,” “poorly,”) or ever having need for medical interpretation at their doctor’s office	Cantonese, Mandarin	Speaking English “poorly” or ‘not at all,’ was significantly associated with poor self-rated health in regression models. The need for a medical interpreter was not associated with self-rated health.

NA means not available in the published manuscript. Abbreviations used in the table include the following: LEP, limited English proficiency; EP, English proficient.

## 4. Discussion

Our comprehensive review of 137 studies on the impact of LEP on healthcare outcomes reveals nuanced patterns across different healthcare settings and conditions. The diversity in study design, participant demographics, and outcome measures necessitates a careful interpretation of findings and highlights the complexities of addressing healthcare disparities among LEP populations. The lack of standardized definitions for LEP and variations in healthcare settings may contribute to inconsistencies in reported outcomes.

The majority of these studies had small LEP sample sizes and considerable variability in defining the LEP population. One of the difficulties our team encountered during searching was the tendency to conflate language interpretation with language translation. Interpretation refers to spoken language, while translation is meant to refer only to written language. However, in practice, these terms are often used interchangeably, and there tends to be some overlap and a lack of precision in their use. In addition, defining LEP varies by type of study and even within type, making it difficult to compare across studies, which may account for differences in outcomes. Studies that included data extracted from electronic health records (EHR) used either the need for an interpreter, which can underreport LEP since not everyone with LEP requests an interpreter, or a flag indicating LEP, which may be inaccurate. A recent study conducted at two hospitals showed positive predictive values as low as 60% for non-English preference in the EHR [154]. Indeed, the study by Balakrishnan et al. found that a significant number of patients who described themselves as Spanish speakers were misclassified as English speakers [75]. In addition, non-native English speakers may have difficulty with medical terminology, creating a language barrier in this particular setting. Issues of misclassification can dilute differences between LEP and EP populations, attenuating what may be potentially significant associations. Studies that included surveys generally used self-report or the language of the survey to determine LEP. Since surveys are often available in limited languages, this can restrict the LEP populations that we can assess for healthcare access and outcomes. Notably, Spanish was overwhelmingly the most studied language group, emphasizing the need for more inclusive language representation in future research. 

The reliance on retrospective chart reviews and cross-sectional designs limits causal inference and underscores the need for prospective and experimental studies. The preponderance of observational studies is understandable, given the complexities and institutional resource demands associated with quasi-experimental and experimental studies. However, the observational nature of these studies limits our ability to establish causal relationships and may introduce confounding variables that independently influence outcomes and are not controlled for in the studies. We saw this in differential outcomes by language group and biological sex, and after modeling, we controlled for socio-demographic characteristics like health insurance and education that may be linked to both immigrant and LEP status. An example of this can be found in the ambulatory care results for the Pylypchuk and Hudson study, which showed differential access to ambulatory care by insurance status [30]. Many studies, particularly those focusing on hospital-based treatments and surgeries, had relatively small LEP patient sample sizes. This limits the generalizability of findings and underscores the importance of conducting studies with larger, more diverse populations to enhance the external validity of the results. The studies covered a spectrum of chronic and acute health issues and settings, ranging from diabetes to mental health conditions and even surgical outcomes. While this diversity enriches our understanding of health disparities, it complicates the task of drawing generalizable conclusions. The varying benchmarks across conditions and healthcare modalities make it challenging to create a unified framework for assessing outcomes, emphasizing the need for interpretations that take into account potential confounding variables.

Importantly, the overall results on healthcare access and outcomes showed that LEP populations experience disparities in access and outcomes across both ambulatory and acute care, as well as in specific condition outcomes and general physical and mental health outcomes:

1. Ambulatory care studies generally showed that the LEP population was less likely to have a usual source of care and had fewer ambulatory care visits.
2. In the hospitalization studies, 30-day readmission was clearly higher for LEP populations, which dovetails with the studies that found that LEP patients were less likely to receive discharge instructions that they could understand and, therefore, less likely to follow through with discharge instructions.
3. Both the specific condition and general health outcome studies show a pattern of worse outcomes for physical and mental health for LEP populations compared to EP populations.

Particularly interesting areas to focus on for further research are those where studies showed mixed outcomes. This could be due to communication barriers, cultural barriers, socio-economic barriers, or a combination of factors. The impact of LEP on LOS was variable by reason for hospitalization, with increased LOS shown for trauma-related and major surgery hospitalizations, but no differences in LOS were found for other types of hospitalizations. Hospitalization outcomes for specific conditions also showed mixed results on the impact of LEP. This may indicate areas where addressing communication barriers can make a difference in outcomes and cost. Colorectal and cervical screenings generally showed that LEP populations were less likely to be screened, meaning that cancer may be found at a later stage, resulting in poor outcomes. However, mammography screening studies were almost evenly split between no effect of LEP and LEP populations accessing fewer screenings. This is a key area that may allow us to explore differences between language groups and even geographic regions in order to identify what is driving differential outcomes. 

## 5. Limitations

One challenge specific to identifying the literature on LEP populations, as we observed in our review, is that there is wide variability in how LEP is defined and how those definitions are applied. There are also a large variety of disciplines investigating this issue from different perspectives. In an effort to cast a wide net, we included very different types of databases, necessitating some variability in our search strings. We acknowledge that this variability has the potential to introduce selection bias. We additionally elected to focus on spoken language only, which excludes any studies focused primarily on written communication, even if these studies might independently show an impact on LEP health outcomes. We also chose not to focus our searches on other closely related topics we encountered, such as health literacy and language concordance, in order to focus on the impact of spoken language on healthcare outcomes. In our opinion, these limitations only underscore the need for further work in this area. 

## 6. Conclusions

Despite the inherent limitations, these findings underscore the urgent need for targeted interventions and policy initiatives in the U.S. to address disparities in conditions ranging from chronic diseases to surgical outcomes for the LEP population. Future research endeavors should prioritize experimental designs, explore the impact of language concordance and translation services, and strive for larger and more diverse sample sizes to enhance the robustness and generalizability of findings. A key part of this will be the consistent definition and collection of LEP data in both EHRs and surveys. Table 2, which includes publicly available datasets used for LEP access and outcome analysis, can be a first step in increasing research in this area. This review provides a foundation for advancing our understanding of health outcomes in LEP populations. By addressing the identified limitations and building on these insights, researchers can contribute to the development of tailored interventions that mitigate health disparities, ultimately promoting equitable healthcare for all linguistic communities.

## Figures and Tables

**Table 1:** Search summary table.

Database	Search String and/or Strategy	Filters	Number of Results
PubMed	((((limited English proficiency[MeSH Terms]) OR (language barrier[MeSH Terms])) AND ((((hospitalization[MeSH Terms]) OR (delivery of healthcare[MeSH Terms])) OR (quality of healthcare[MeSH Terms])) OR (emergency departments[MeSH Terms]))) AND (united states)) NOT (digital divide[MeSH Terms])	English Exclude preprints Adult: 19+ years From 2000 to 2023	935
Academic Search Premier	SU (“limited English proficiency” OR “language barriers”) AND SU (“health services accessibility” OR “health outcome assessment” OR “hospitals” OR “emergency departments”) NOT SU (“digital literacy” OR “digital divide”)	SmartText Searching English Source Type(s): Academic Journals and Trade Publications From 2000 to 2023	292
CINAHL	((((limited English proficiency) OR (language barrier)) AND ((((hospitalization) OR (delivery of healthcare)) OR (quality of healthcare)) OR (emergency departments))) AND (united states)) NOT (digital divide)	Basic Search English Source Type(s): Academic Journals and Dissertations From 2000 to 2023	326
Sociological Abstracts	(“limited English proficiency” OR “language barriers” OR “language proficiency”) AND (health care)	Advanced Search English Source Types: Scholarly Journals, Dissertations and Theses, and Other Sources. United States From 2000 to 2023	956
EconLit	TX language proficiency AND SU (“health care” OR “health behavior”)	English Source Type(s): Academic Journals and Dissertations From 2000 to 2023	16
TOTAL	-	-	2525

**Table 2:** Publicly available datasets with information on LEP used by studies in this scoping review.

Publicly Available State or National Dataset Used	Research Focus Area	Number of Papers Using
American Hospital Association (AHA) Annual Survey	Hospital Care	1
Asian American Quality of Life Survey	Ambulatory Care	1
Behavioral Risk Factor Surveillance System	Screening	3
California Cancer Registry	Specific Conditions	1
California Health Interview Survey	Ambulatory Care; General Health Outcomes; Screening	10
Community Tracking Study Household Survey	Ambulatory Care	1
DISTANCE Study Data (Diabetes Study of Northern California)	Specific Conditions	3
Hawaii’s Health Information Corporation’s Inpatient Database	Hospital Care	1
Hispanic Established Population for Epidemiological Studies of the Elderly (Hispanic EPESE)	Specific Conditions	2
Los Angeles Latino Eye Study (LALES)	Ambulatory Care	1
Medical Expenditure Panel Survey	Ambulatory Care	2
Minnesota Community Measures registry	Specific Conditions	1
National Agricultural Workers Survey (NAWS)	Ambulatory Care	1
National Consumer Assessment of Healthcare Providers and Systems (CAHPS) Benchmarking Database (NCBD)	Ambulatory Care	1
National Health and Aging Trends Study	Specific Conditions	1
National Health and Nutrition Examination Survey (NHANES)	Specific Conditions	2
National Health Interview Survey (NHIS)	Ambulatory Care	2
National Latino and Asian American Household Survey	Ambulatory Care; General Health Outcomes	3
National Trauma Registry of the American College of Surgeons (NTRACS)	Hospital Care	1
New Immigrant Survey	General Health Outcomes	1
State Inpatient Database (SID)—New Jersey *	Specific Conditions	1
State Inpatient Database (SID)—California *	Hospital Care	1
Study of Women’s Health Across the Nation (SWAN)	Screening	1

* Currently only 7 states have patient language data available in their SID.

## Data Availability

No new data were created or analyzed in this study. Data sharing is not applicable to this article.

## Supplementary Materials

The following supporting information can be downloaded at: https://www.mdpi.com/article/10.3390/healthcare12030364/s1, Preferred Reporting Items for Systematic reviews and Meta-Analyses extension for Scoping Reviews (PRISMA-ScR) Checklist. Ref. [155] is cited in Supplementary Materials.
